# Hypertension promotes bone loss and fragility by favoring bone resorption in mouse models

**DOI:** 10.1172/JCI184325

**Published:** 2025-08-19

**Authors:** Elizabeth M. Hennen, Sasidhar Uppuganti, Néstor de la Visitación, Wei Chen, Jaya Krishnan, Lawrence A. Vecchi, David M. Patrick, Mateusz Siedlinski, Matteo Lemoli, Rachel Delgado, Mark P. de Caestecker, Wenhan Chang, Tomasz J. Guzik, Rachelle W. Johnson, David G. Harrison, Jeffry S. Nyman

**Affiliations:** 1 Department of Biomedical Engineering, Vanderbilt University, Nashville, Tennessee, USA.; 2 Department of Veterans Affairs, Tennessee Valley Healthcare System, Murfreesboro, Tennessee, USA.; 3 Department of Orthopedic Surgery, Department of Medicine, and; 4 Division of Clinical Pharmacology, Department of Medicine, Vanderbilt University Medical Center, Nashville, Tennessee, USA.; 5 Centre for Cardiovascular Sciences, University of Edinburgh, Edinburgh, United Kingdom.; 6 Department of Internal and Agricultural Medicine and Omicron Medical Genomics Laboratory, Jagiellonian University, Collegium Medicum, Krakow, Poland.; 7 Department of Clinical and Experimental Sciences, University of Brescia, Brescia, Italy.; 8 Division of Nephrology and Hypertension, Department of Medicine, Vanderbilt University Medical Center, Nashville, Tennessee, USA.; 9 San Francisco VA Medical Center, Department of Medicine, University of California, San Francisco, San Francisco, California, USA.

**Keywords:** Bone biology, Immunology, Inflammation, Bone disease, Bone marrow, Hypertension

## Abstract

Inflammatory diseases contribute to secondary osteoporosis. Hypertension is a highly prevalent inflammatory condition that is clinically associated with reduced bone mineral density and increased risk of fragility fracture. In this study, we showed that a significant loss in bone mass and strength occurs in two preclinical models of hypertension. This accompanied increases in immune cell populations, including monocytes, macrophages, and IL-17A–producing T cell subtypes in the bone marrow of hypertensive mice. Neutralizing IL-17A in angiotensin II–infused mice blunted hypertension-induced loss of bone mass and strength as a result of decreased osteoclastogenesis. Likewise, the inhibition of the CSF1 receptor blunted loss of bone mass and prevented loss of bone strength in hypertensive mice. In an analysis of UK Biobank data, circulating bone remodeling markers exhibited striking associations with blood pressure and bone mineral density in more than 27,000 humans. These findings illustrate a potential mechanism by which hypertension activates immune cells in the bone marrow, encouraging osteoclastogenesis and eventual loss in bone mass and strength.

## Abstract 2

We show that hypertension promotes bone loss and formation of osteoclasts, and that this is dependent on IL17A and CSF1R signaling. In humans, markers of bone turnover correlate with blood pressure. We conclude that the bone is a target of hypertensive end organ damage.

## Graphical abstract

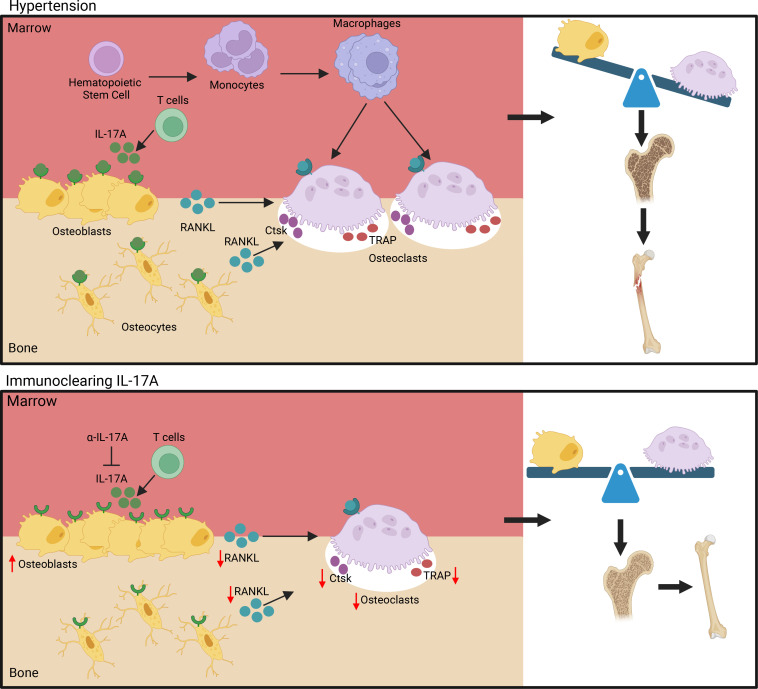

## Introduction

Approximately 200 million people have osteoporosis, and one-third of women and one-fifth of men over 50 years of age experience low-energy fragility fractures (1). Lifestyle- and health-related conditions such as age, physical inactivity, diabetes, tobacco use, autoimmune diseases, and menopause increase the risk of low bone mass and fragility fractures (1, 2). Like osteoporosis, the prevalence of hypertension increases with these conditions. Clinical studies also suggest that hypertension is associated with fragility fractures. Hypertensive patients exhibit lower bone mineral density (BMD) and a greater propensity for fragility fractures (3–6). Additionally, the severity of hypertension correlates with lower hip bone mass in postmenopausal women (7).

Emerging evidence indicates that hypertension is associated with immune activation. Both innate and adaptive immune cells have been observed in the kidneys and blood vessels of hypertensive humans, and either genetic deletion or immunoclearing of cytokines including interferon-γ (IFN-γ) (8), tumor necrosis factor-α (TNF-α) (9), and interleukin-17A (IL-17A) (10, 11) reduces hypertension in experimental models. Likewise, deletion of myeloid cells including macrophages, dendritic cells, and monocytes prevents experimental hypertension (12–14).

Recent studies have emphasized the role of immune activation, and particularly cytokines like IL-17A, in bone remodeling. Huang et al. showed that IL-17A supports osteoclastogenesis by stimulating mesenchyme-derived cells like osteoblasts and osteocytes to produce pro-osteoclastic cytokines (15). Li et al. revealed that deleting the IL-17A receptor (IL-17RA) from mature osteoblasts and osteocytes inhibits IL-17A/IL-17RA signaling, reduces production of receptor activator of nuclear factor-κB ligand (RANKL), and inhibits osteoclast formation and bone resorption (16). It is unknown whether hypertension-induced bone loss is due to an increase in IL-17A.

Preclinical studies as early as 1985 found that spontaneously hypertensive rats (SHRs) have lower bone mass in the femur and tibia than control rats (17). More recent studies indicate that the SHR has reduced bone mass and cortical strength (18). Treatment of SHRs with angiotensin-converting enzyme inhibitors improved BMD and decreased osteoclast number (19). SHRs treated with amlodipine had a dose-dependent increase in bone density with a reduction of circulating markers for osteoclast activity (20). These studies speak to a potential causative relationship between hypertension and osteoporosis; however, the molecular relationship between these diseases and specific therapeutic interventions has not been clearly defined.

We hypothesized that hypertension shares common etiological features with osteoporosis, causing both the bone and bone marrow to favor osteoclastogenesis, bone resorption, and bone fragility. To test this hypothesis, we performed a rigorous evaluation of bone mass, strength, and remodeling and bone marrow immune cells. We examined how immune modulation during angiotensin II (Ang II) hypertension blunts hypertension-induced bone loss. To provide translational proof of concept, we examined plasma markers of bone turnover in humans in the UK Biobank population.

## Results

### Hypertension causes a systemic loss of bone mass and strength in two models of hypertension.

**Figure 1:**
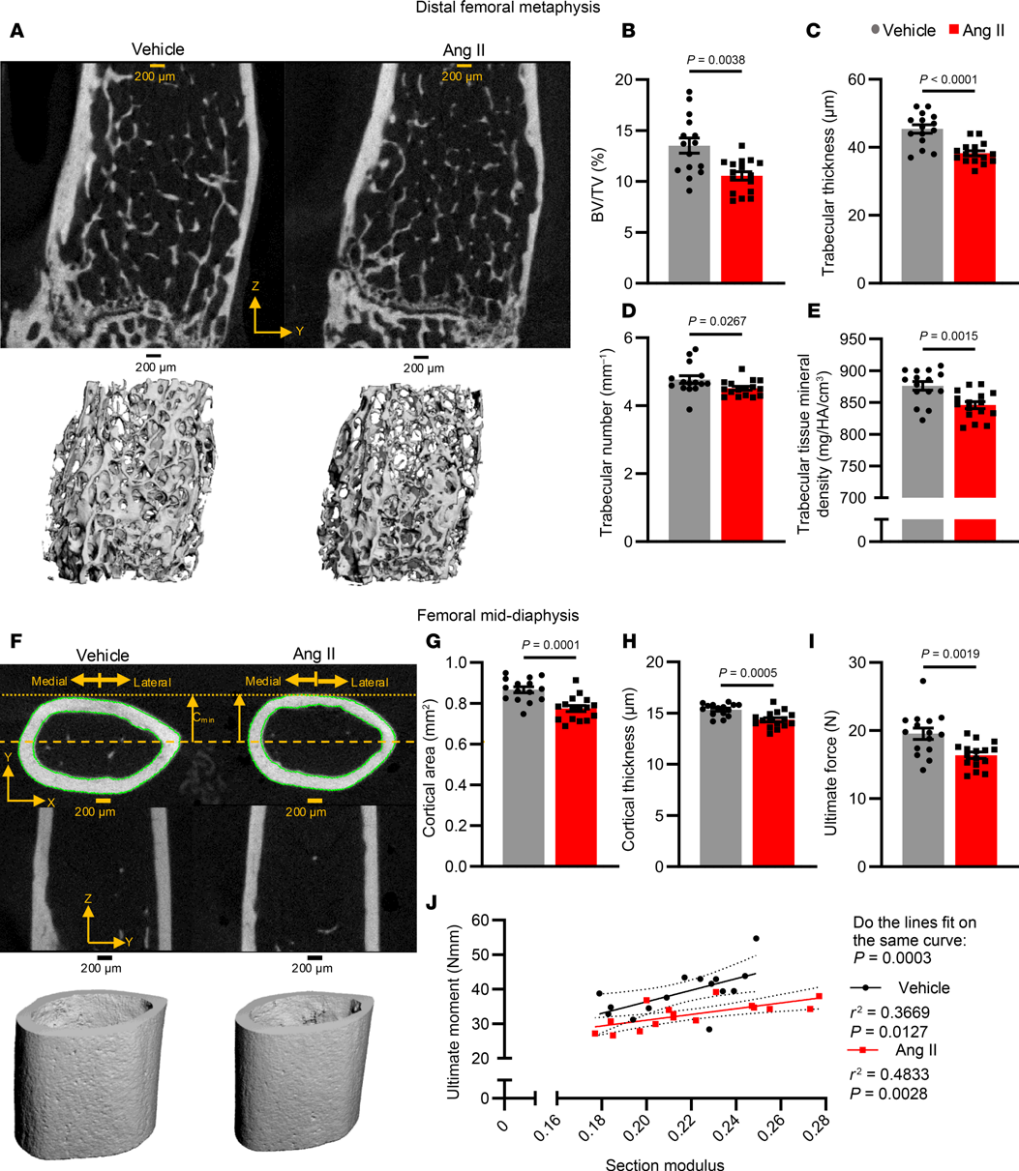
Trabecular architecture of the distal femoral metaphysis and cortical structure of the femoral mid-diaphysis from vehicle- and Ang II–infused mice. (**A**) Representative images of 2D (top) and 3D (bottom) renderings of the metaphysis. (**B**–**F**) μCT-derived parameters, including trabecular BV/TV (**B**), thickness (**C**), number (**D**), and tissue mineral density (**E**). (**F**) Representative images of 2D (top) and 3D (bottom) rendering of the diaphysis. (**G** and **H**) μCT-derived parameters, including cortical area (**G**) and thickness (**H**). (**I**) The ultimate force from 3-point bending. (**J**) Correlation of section modulus and ultimate moment (Newton*millimeters, Nmm) for vehicle-infused (black) and Ang II–infused (red) mice. **C**, **E**, **G**, and **H** were analyzed by unpaired *t* test. **B**, **D**, and **I** were analyzed by Mann-Whitney test. **J** was analyzed by nonlinear regression. SEM is shown. Sample size: Vehicle, *n* = 15; Ang II, *n* = 16. Scale bars: 200 μm.

To define the effect of hypertension on bone mass and bone quality, we first induced hypertension in mice by Ang II infusion for 6 weeks. We confirmed that the systolic blood pressure (SBP) rose to approximately 160 mmHg in this model without changes in heart rate, using noninvasive tail-cuff measurements (Supplemental Table 1; supplemental material available online with this article; https://doi.org/10.1172/JCI184325DS1). As shown in the example images of the distal femoral metaphysis (Figure 1A) and the comparison of measurements derived from high-resolution micro–computed tomography (μCT) (Figure 1B), Ang II–infused mice had significantly lower bone volume fraction (ratio of bone volume to total volume; BV/TV) compared with vehicle-infused mice. This was due to reduced trabecular thickness (Figure 1C) and number (Figure 1D), and resulted in higher separation between trabeculae (Supplemental Table 2). Femora from hypertensive mice also had decreased trabecular tissue mineral density compared with those from normotensive mice (Figure 1E). The femoral mid-diaphysis was scanned to assess differences in cortical structure (Figure 1F). Hypertensive mice had reduced cortical area (Figure 1G) and thickness (Figure 1H) compared with vehicle-infused mice. Ang II and vehicle mice had no differences in other cortical properties (Supplemental Table 2). Three-point bending studies showed that the femora from Ang II–treated mice withstood significantly lower ultimate force (Figure 1I) and exhibited other mechanical parameters compatible with loss of bone strength (Supplemental Table 2). For a given section modulus (a measure of structural resistance to bending), the ultimate moment (ultimate force × span/4) endured during 3-point bending was significantly lower in the Ang II–infused mice compared with the vehicle-infused mice (Figure 1J). This suggests a structure-independent change in cortical strength with Ang II infusion.

**Figure 2:**
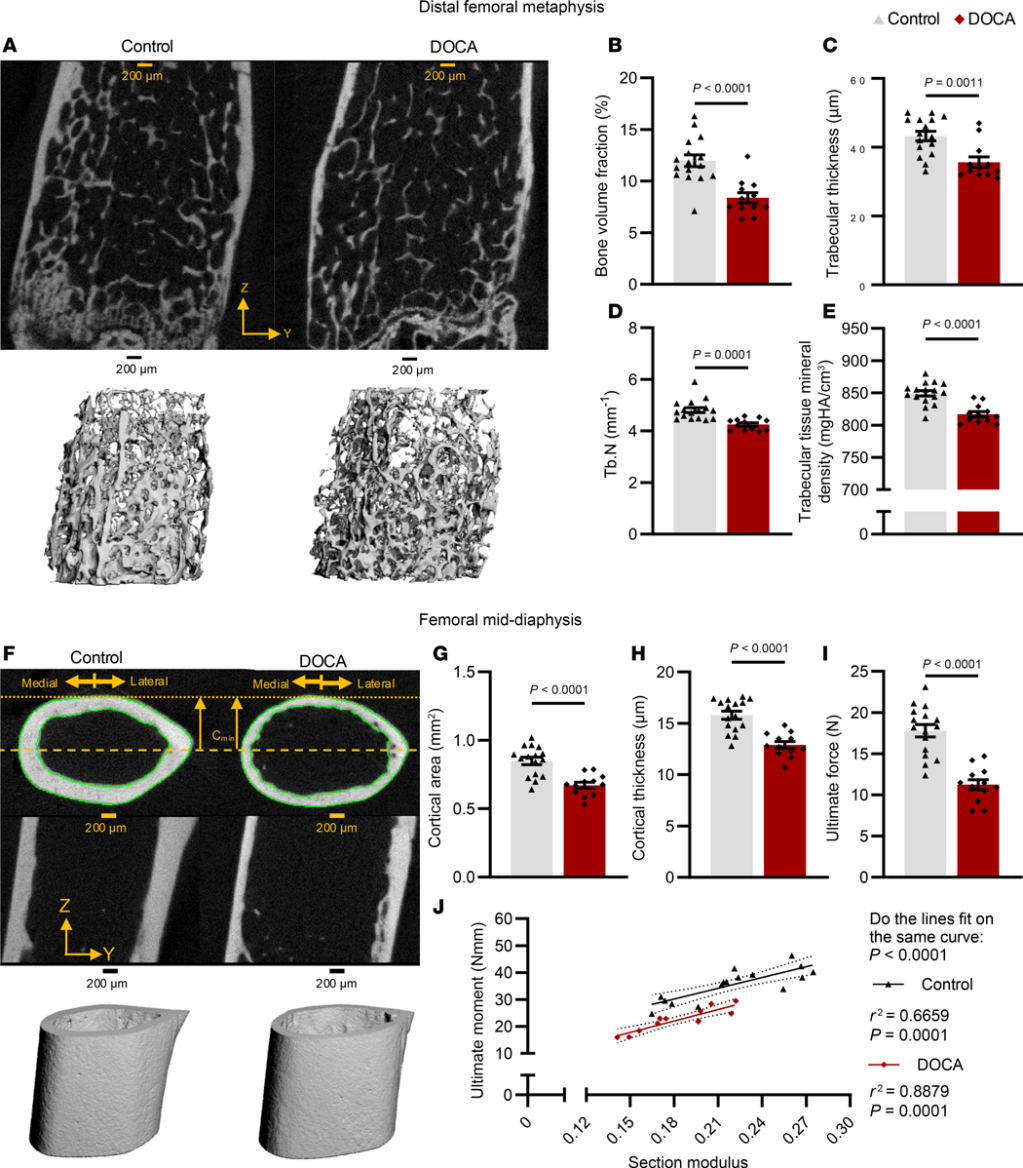
Trabecular architecture of the distal femoral metaphysis and cortical structure of the femoral mid-diaphysis from control and DOCA-salt mice. (**A**) Representative images of 2D (top) and 3D (bottom) renderings of the metaphysis. (**B**–**F**) Quantification of trabecular BV/TV (**B**), thickness (**C**), number (**D**), and tissue mineral density (**E**). (**F**) Representative images of 2D (top) and 3D (bottom) rendering of the diaphysis. (**G** and **H**) Quantification of cortical area (**G**) and thickness (**H**). (**I**) Ultimate force. (**J**) Correlation between section modulus and ultimate moment for control (black) and DOCA-salt (red) mice. **C**, **D**, and **F**–**H** were analyzed by unpaired *t* test. **B**, **E**, and **I** were analyzed by Mann-Whitney test. **J** was analyzed by nonlinear regression. SEM is shown. Sample size: Control, *n* = 16; DOCA, *n* = 12. Scale bars: 200 μm.

To determine whether alterations in bone mass and strength are specific to Ang II–induced hypertension, we performed additional studies in mice with deoxycorticosterone acetate (DOCA)–salt hypertension (11). In this model, circulating Ang II levels are suppressed (11). Like the Ang II model, mice subjected to DOCA-salt–induced hypertension exhibited significant elevation in SBP with no change in heart rate (Supplemental Table 1). As in Ang II–induced hypertension, metaphyseal BV/TV was reduced by DOCA-salt hypertension in comparison with control mice that had received only nephrectomy and a control pellet (Figure 2, A and B). This was reflected by a reduction in trabecular thickness (Figure 2C) and number (Figure 2D), and an increase in separation (Supplemental Table 2). DOCA-salt hypertension also reduced trabecular tissue mineral density (Figure 2E). DOCA-salt hypertension had no effect on trabecular connectivity density but did increase the structure index in the metaphysis, reflecting a less optimal trabecular shape (Supplemental Table 2). Like in the Ang II model, mice with DOCA-salt hypertension had reduced cortical area (Figure 2, F and G) and thickness (Figure 2, F and H). There were no significant differences in cortical tissue mineral density, volumetric BMD, and porosity between hypertensive and normotensive mice (Supplemental Table 2). Similarly to Ang II–induced hypertension, femora from mice with DOCA-salt hypertension exhibited reduced ultimate force during 3-point bending (Figure 2I). The best-fit lines for the section modulus versus the ultimate moment had a similar slope but were significantly offset between the two groups (Figure 2J).

We also used other μCT measures to characterize differences in bone shape and size between normotensive and hypertensive mice. Neither model showed significant changes in cross-sectional characteristics of the mid-diaphysis such as total area and minimum moment of inertia (Supplemental Table 2). There was a significant decrease in polar moment of inertia and increase in medullary volume in the DOCA-salt model only (Supplemental Table 2). The lack of a hypertensive effect on minimum moment of inertia was corroborated by an absence of noticeable changes in the anterior-posterior diameter in either model (Supplemental Table 2). While mice with Ang II–induced hypertension had significantly shorter femora than vehicle-treated mice, no other significant structural changes were observed (Supplemental Table 2).

Vertebral bodies (VBs) of the spine are common sites of osteoporosis and compression fractures, often due to the loss of trabecular bone (21). Therefore, we examined bone mass and strength of the sixth lumbar (L6) VB. There was a significant reduction in BV/TV in the L6 VB of Ang II–infused mice (Supplemental Figure 1, A and B) due to a reduction in trabecular thickness but not trabecular number (Supplemental Figure 1, C and D). Trabecular tissue mineral density was reduced in the L6 VB of Ang II–infused mice (Supplemental Figure 1E). To assess the strength of L6 VB from each group, 2 methods were used. μCT-derived, linear elastic finite element analysis (μFEA), which estimates the compressive force needed to cause L6 VB to fail, was significantly decreased in Ang II–infused mice (Supplemental Figure 1F). Subsequent compression testing confirmed that the ultimate force withstood by L6 VB was reduced in Ang II–infused mice (Supplemental Figure 1G). DOCA-salt hypertension caused similar reductions in BV/TV, trabecular thickness, tissue mineral density, estimated failure load, and ultimate force (Supplemental Figure 1, H–J and L–N), but also decreased trabecular number (Supplemental Figure 1K). Both models of hypertension similarly affected other parameters of trabecular architecture and strength (Supplemental Table 3).

### Alterations in bone quality caused by hypertension.

Knowing that the hypertension-related decrease in bending strength of cortical bone was independent of bone structure, we investigated the effect of hypertension on tissue hydration. Water bound to the bone matrix is an important component of bone strength and reflects the quality of the matrix in that higher bound water levels indicate stronger, tougher bone (22). We used ^1^H nuclear magnetic resonance (^1^H-NMR) relaxometry to quantify bound water in the femora. There was a decline in bound water in the femora of DOCA-salt mice compared with their controls only (Supplemental Figure 2, A and B). Alterations in bone quality such as this are often accompanied by increased risk for fracture (22, 23). We therefore examined fracture toughness (i.e., the ability of cortical bone to resist crack growth) and found no difference between Ang II– and vehicle-infused mice (Supplemental Figure 2C). Conversely, there was a considerable reduction in fracture toughness between control and DOCA-salt–treated mice (Supplemental Figure 2D).

Kidney disease is a known determinant of bone fragility and increases the risk for fractures (24). Therefore, we calculated glomerular filtration rates (GFRs) by measuring the decay of a fluorescently conjugated inulin analog using a transdermal detector, transdermal GFR (tGFR), as previously described (25), in both models of hypertension. Ang II infusion caused no changes in tGFR, while DOCA-salt hypertension caused a marginal decrease (*P* = 0.0559) in tGFR (Supplemental Figure 2, E and F). Parathyroid hormone (PTH) dysregulation augments kidney disease–bone mineralization disorder (26). PTH levels were not affected by Ang II–induced hypertension but were increased by 25% in DOCA-salt hypertension (Supplemental Figure 2, G and H).

### Hypertension enhances myelopoiesis and osteoclastogenesis.

**Figure 3:**
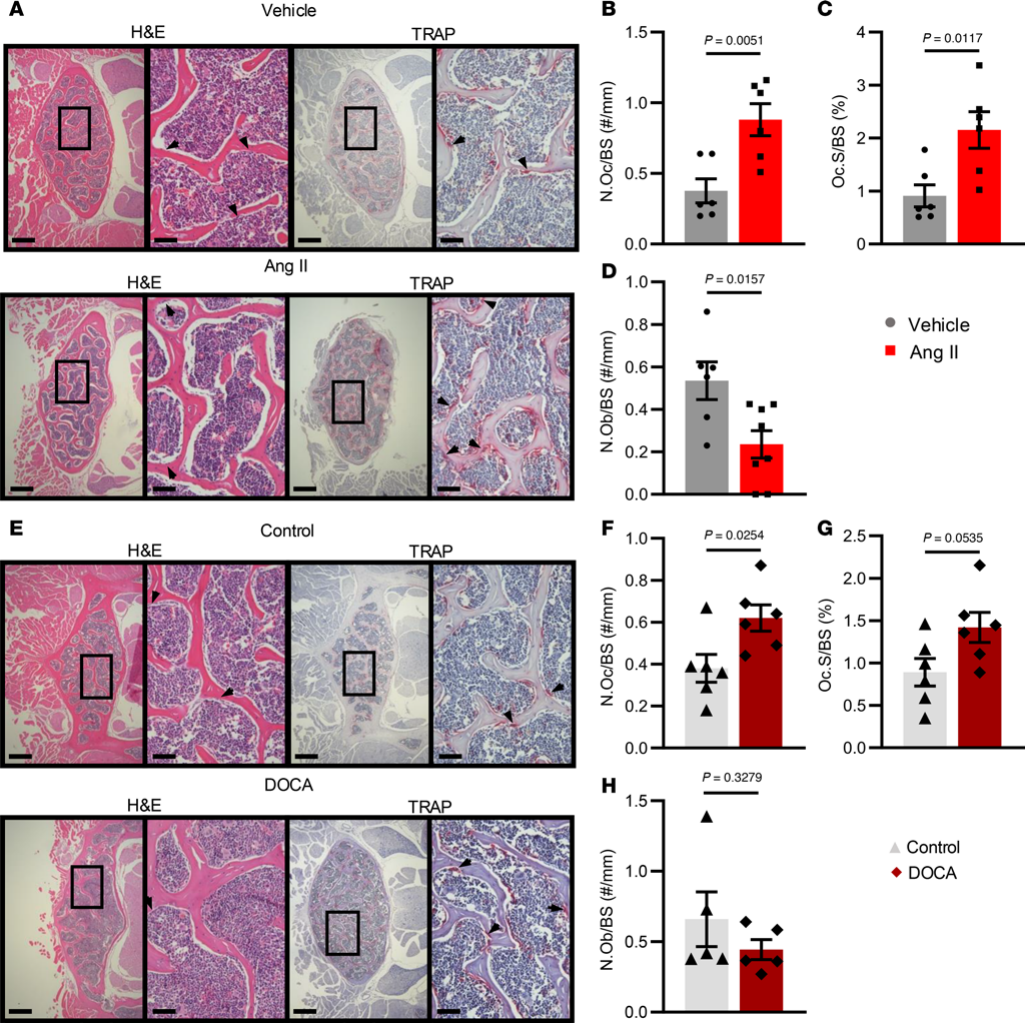
Histological analysis of hematoxylin and eosin– and TRAP-stained sections of the lumbar spine. (**A** and **E**) Representative histological images. Hematoxylin and eosin–stained (H&E-stained) and TRAP-stained sections are shown at 2 views (original magnification, ×4 at left and ×20 at right). Left scale bars: 200 μm; right scale bars: 50 μm. (**B** and **F**) Quantification of the number of osteoclasts (N.Oc) divided by bone surface (BS) from both models of hypertension. (**C** and **G**) Quantification of the osteoclast surface (Oc.S) divided by BS from both models of hypertension. (**D** and **H**) Quantification of the number of osteoblasts (N.Ob) divided by BS from both models of hypertension. Unpaired *t* test was used. SEM is shown. Sample size: Vehicle, *n* = 6; Ang II, *n* = 6–8; control, *n* = 6–8; DOCA, *n* = 6–8.

Bone remodeling is a dynamic process in which bone formation and bone resorption activity are coupled. Bone loss is commonly associated with increased bone resorption caused by elevated osteoclast number or activity, and/or decreased bone formation caused by lower osteoblast number or activity. Using tartrate-resistant acid phosphatase (TRAP) staining of formalin-fixed paraffin-embedded sections from the third and fourth VBs, we observed a significant increase in TRAP^+^ osteoclast number (N.Oc) and surface (Oc.S) normalized to the bone surface (BS) during Ang II–induced hypertension (Figure 3, A–C). Additionally, Ang II–infused mice had lower osteoblast numbers (N.Ob) normalized to BS (Figure 3D). DOCA-salt hypertension caused a significant increase in N.Oc/BS and a moderate increase in Oc.S/BS (Figure 3, G–I). We observed no difference in N.Ob/BS between control and DOCA-salt–treated mice (Figure 3J).

**Figure 4:**
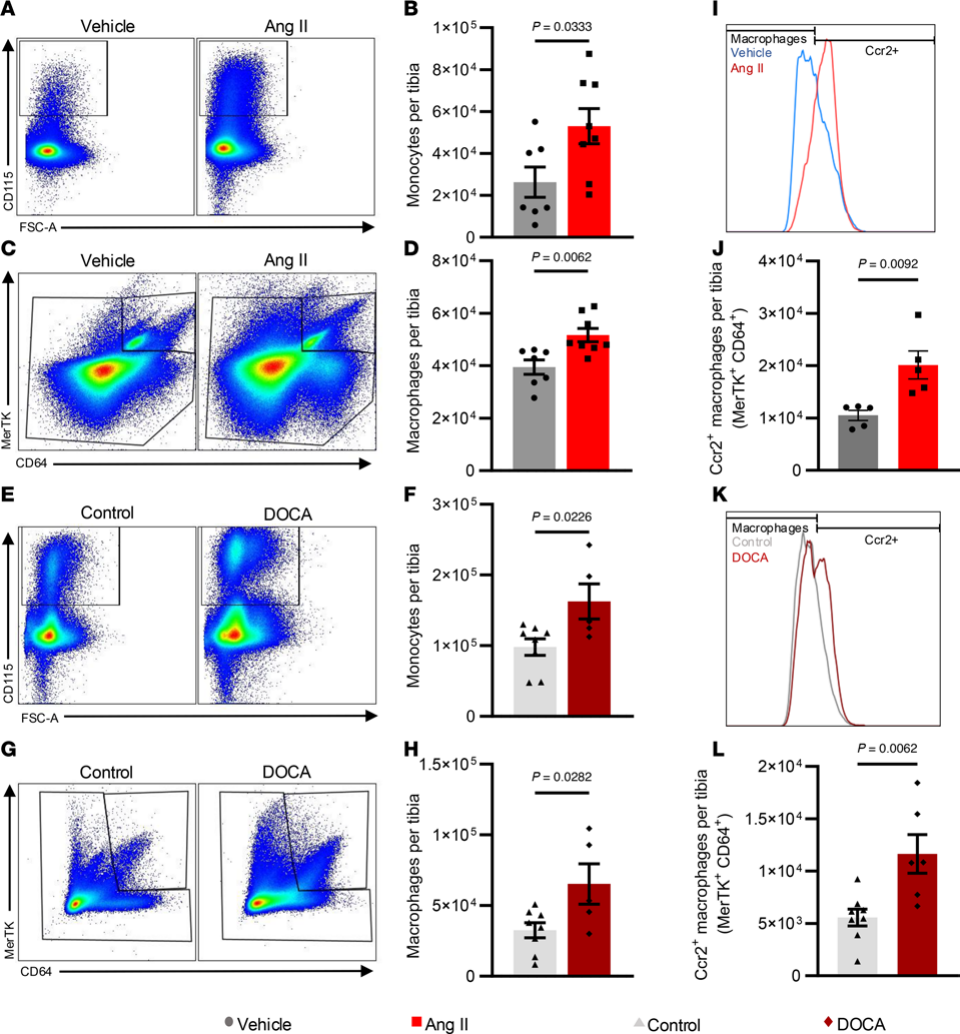
Flow cytometric analysis of the bone marrow in both models of hypertension for monocytes and macrophages. (**A** and **B**) Representative gating for monocytes (**A**) and quantification of monocytes (**B**) from vehicle- and Ang II–infused mice. (**C** and **D**) Representative gating for macrophages (**C**) and quantification of macrophages (**D**) from vehicle- and Ang II–infused mice. (**E**–**H**) Gating and quantification of monocytes and macrophages in the bone marrow of control and DOCA mice, respectively. (**I** and **J**) Quantification of Ccr2^+^ macrophages in the bone marrow of vehicle- and Ang II–infused mice. (**K** and **L**) Quantification of Ccr2^+^ macrophages in the bone marrow of control and DOCA mice. Unpaired *t* test was used. SEM is shown. Sample size: Vehicle, *n* = 5–7; Ang II, *n* = 5–8; control, *n* = 8; DOCA, *n* = 5–6.

Osteoclasts are generally considered products of myelopoiesis, arising from myeloid precursors. Therefore, we examined myeloid cells in the bone marrow of normotensive and hypertensive mice. In Ang II–induced and DOCA-salt hypertension, monocytes (Figure 4, A, B, E, and F) and macrophages (Figure 4, C, D, G, and H) were significantly increased in the bone marrow. We also quantified C-C chemokine receptor type 2–positive (CCR2^+^) macrophages, which are recognized as bone marrow derived (27), and found that these were likewise increased in both models of hypertension (Figure 4, I–L).

**Figure 5:**
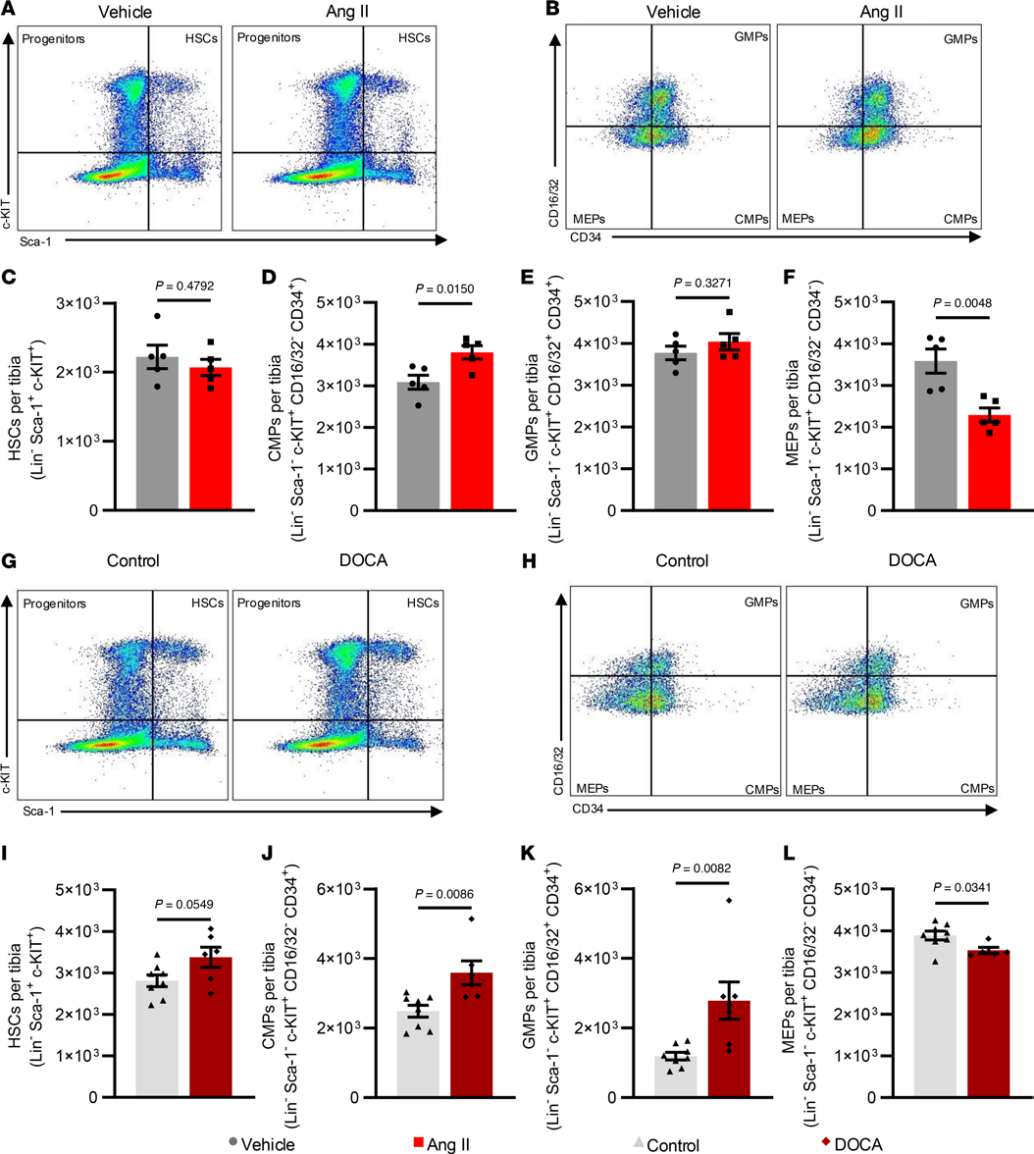
Flow cytometric analysis of bone marrow progenitor populations in both models of hypertension. (**A**, **B**, **G**, and **H**) Representative flow gates for HSCs (**A** and **G**) and myeloid progenitors (**B** and **H**). (**C** and **I**) Quantification of HSCs. (**D** and **J**) Quantification of CMPs. (**E** and **K**) Quantification of GMPs. (**F** and **L**) Quantification of MEPs. **C**–**F**, **I**, **K**, and **L** were analyzed by unpaired *t* test. Mann-Whitney test was used for **J**. SEM is shown. Sample size: Vehicle, *n* = 5; Ang II, *n* = 5; control, *n* = 8; DOCA, *n* = 6.

Previous work showed that hypertension promotes expansion of hematopoietic progenitor populations in the bone marrow (28). Therefore, we quantified hematopoietic stem cells (HSCs), common myeloid progenitors (CMPs), granulocyte-monocyte progenitors (GMPs), and megakaryocyte-erythrocyte progenitors (MEPs) in the bone marrow of normotensive and hypertensive mice. CMPs give rise to both GMPs and MEPs; GMPs can become granulocytes or non-granulocyte innate immune cells, whereas MEPs give rise to megakaryocytes or erythrocytes. Vehicle- and Ang II–infused mice had comparable numbers of HSCs and GMPs; however, Ang II–infused mice exhibited increased numbers of CMPs (Figure 5, A–D) and decreased MEPs (Figure 5, A, B, E, and F). DOCA-salt–treated mice had marginally increased HSCs (*P* = 0.0549) and significant increases in CMPs and GMPs (Figure 5, G–K). MEPs significantly decreased in DOCA-salt–treated mice compared with controls (Figure 5, H and L).

**Figure 6:**
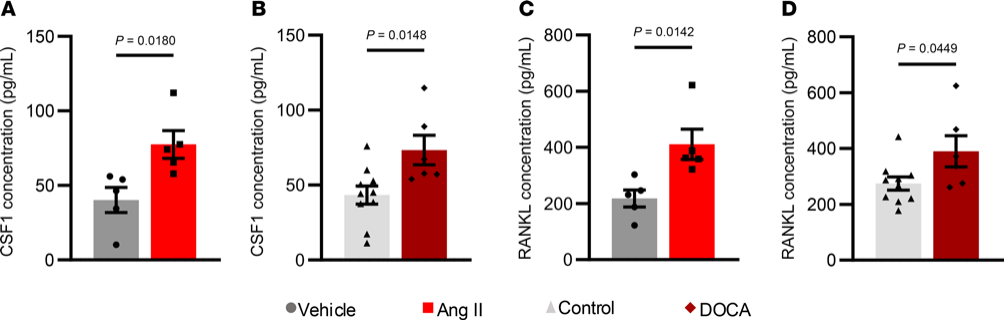
Concentration of pro-osteoclastic cytokines in the bone marrow from both models of hypertension. (**A** and **B**) The concentration of CSF1 in the bone marrow from the Ang II model (**A**) or DOCA-salt model (**B**). (**C** and **D**) The concentration of RANKL in the bone marrow from the Ang II model (**C**) or DOCA-salt model (**D**). All panels were analyzed using unpaired *t* tests. Sample size: Vehicle, *n* = 5; Ang II, *n* = 5; control, *n* = 10; DOCA, *n* = 6.

Colony-stimulating factor 1 (CSF1) promotes the formation of myeloid progenitors, monocytes, macrophages, and osteoclasts. Because hypertension seemed to drive myelopoiesis, we additionally quantified CSF1 levels in the bone marrow of these mice. The concentration of CSF1 (Figure 6, A and B) was increased in the bone marrow of both Ang II–infused mice and DOCA-salt mice compared with their respective controls. RANKL plays a critical role in the formation of osteoclasts from myeloid precursors, and we observed higher bone marrow concentrations of this cytokine in both models (Figure 6, C and D).

### Transcriptional analysis in the bone and bone marrow revealed pro-osteoclastic phenotype.

To examine mechanisms underlying increased osteoclast formation in hypertension, we examined transcriptomic changes in the bone and bone marrow. In marrow cells flushed from humeral bones of Ang II–treated mice, we observed increased mRNA expression of cathepsin K (*Ctsk*) and TRAP (*Acp5*), which are expressed by osteoclasts and serve as markers of osteoclast resorptive activity (Supplemental Figure 3A). Concurrently, there was an increase in colony-stimulating factor 1 receptor (*Csf1r*) and nuclear factor of activated T cells 1 (*Nfatc1*), which dictate pre-osteoclast formation and osteoclast differentiation (Supplemental Figure 3A). We likewise examined RNA transcripts in homogenates of pulverized humeral bone without bone marrow and found a significant increase in expression of CSF1 (*Csf1*) and RANKL (*Tnfsf11*), cytokines needed for pre-osteoclast and osteoclast differentiation, respectively, in mice with Ang II–induced hypertension (Supplemental Figure 3B). We observed no change in osteoprotegerin (OPG; *Tnfrsf11b*), a competitive receptor for RANKL that suppresses osteoclastogenesis (Supplemental Figure 3B). Interestingly, *Ctsk* and *Acp5* (TRAP) expression was significantly increased in the bone of Ang II–treated mice compared with vehicle-treated mice (Supplemental Figure 3B). The ratio of *Tnfsf11* (RANKL) and *Tnfrsf11b* (OPG) was significantly higher in Ang II–infused mice compared with vehicle-infused mice (Supplemental Figure 3C).

Like bone marrow cells from Ang II–infused mice, bone marrow cells from DOCA-salt–treated mice had significantly increased mRNA expression of *Ctsk*, *Acp5* (TRAP), *Csf1r*, and *Nfatc1* compared with controls (Supplemental Figure 3D). Additionally, DOCA-salt–treated mice had increased mRNA expression of *Csf1* and *Tnfsf11* (RANKL) in the bone compared with controls (Supplemental Figure 3E). *Tnfrsf11b* (OPG) mRNA levels were not significantly different in the bone of mice with DOCA-salt hypertension (Supplemental Figure 3E). As in Ang II–induced hypertension, DOCA-salt–treated mice exhibited significant increases in *Ctsk* and *Acp5* (TRAP) mRNA expression in the bone (Supplemental Figure 3E). The ratio of *Tnfsf11* (RANKL) and *Tnfrsf11b* (OPG) was not significantly different with DOCA-salt hypertension (Supplemental Figure 3F).

Transcripts for osteoblast formation and activation in the bone were also quantified. Runt-related transcription factor 2 (*Runx2*), a master regulator of pre-osteoblast differentiation, was significantly decreased in Ang II–infused mice compared with vehicle-infused mice (Supplemental Figure 3G). There was no difference in dentin matrix acidic phosphoprotein 1 (*Dmp1*), a marker for mature osteoblasts and osteocytes, as well as markers for osteoblast activation such as alkaline phosphatase (*Alpl*) and collagen I type 1a (*Col1a1*) (Supplemental Figure 3G). Sclerostin (*Sost*), an inhibitor of osteoblast activity, was not different between vehicle- and Ang II–infused mice (Supplemental Figure 3G). There was a significant reduction in *Runx2* and no change in *Dmp1* in the bone of DOCA-treated mice compared with controls (Supplemental Figure 3H), as found in the Ang II–treated mice. Interestingly, *Alpl* and *Col1a1* in the bone were significantly increased in DOCA-salt–treated mice (Supplemental Figure 3H), indicative of an increase in osteoblast activity. Bone *Sost* mRNA expression was not changed in DOCA-salt hypertension (Supplemental Figure 3H).

In addition to these targeted analyses of mRNA expression, we performed unbiased transcriptomic analyses of bone and bone marrow in Ang II–infused and DOCA-salt hypertensive mice and their respective controls using NanoString nCounter technology (Supplemental Table 4 and Supplemental Figure 4). A general pattern in both bone and marrow for both forms of hypertension was enrichment of genes involved in osteoblast signaling (transforming growth factor-β and wingless-related integration site signaling, proteoglycans, PTH pathway), immune activation (cytokine–cytokine receptor interaction, hematopoietic cell lineage, IL-17A signaling), and osteoclast differentiation (Supplemental Figure 4).

### Serum markers for bone remodeling reflect a pro-osteoclastic phenotype in hypertension.

The serum markers of carboxy-terminus cross-links (CTX-1) and TRAP are markers for bone reabsorption, while procollagen type 1 propeptide (P1NP) is a marker for bone formation. We found increased concentrations of CTX-1 and TRAP in mice with both Ang II–induced and DOCA-salt hypertension, while P1NP levels were unchanged (Supplemental Figure 5, A–F). Because of the coupled nature of bone metabolism, we examined the correlation between CTX-1 and P1NP. In vehicle-infused mice, controls for the DOCA-salt hypertension model, and mice with DOCA-salt hypertension, there was a positive relationship between CTX-1 and P1NP (Supplemental Figure 5, G and H), indicating coupled osteoblastic and osteoclastic activity. Interestingly, in Ang II–infused mice, CTX-1 was elevated irrespective of P1NP levels (Supplemental Figure 5G).

### IL-17A orchestrates bone loss in hypertension.

**Figure 7:**
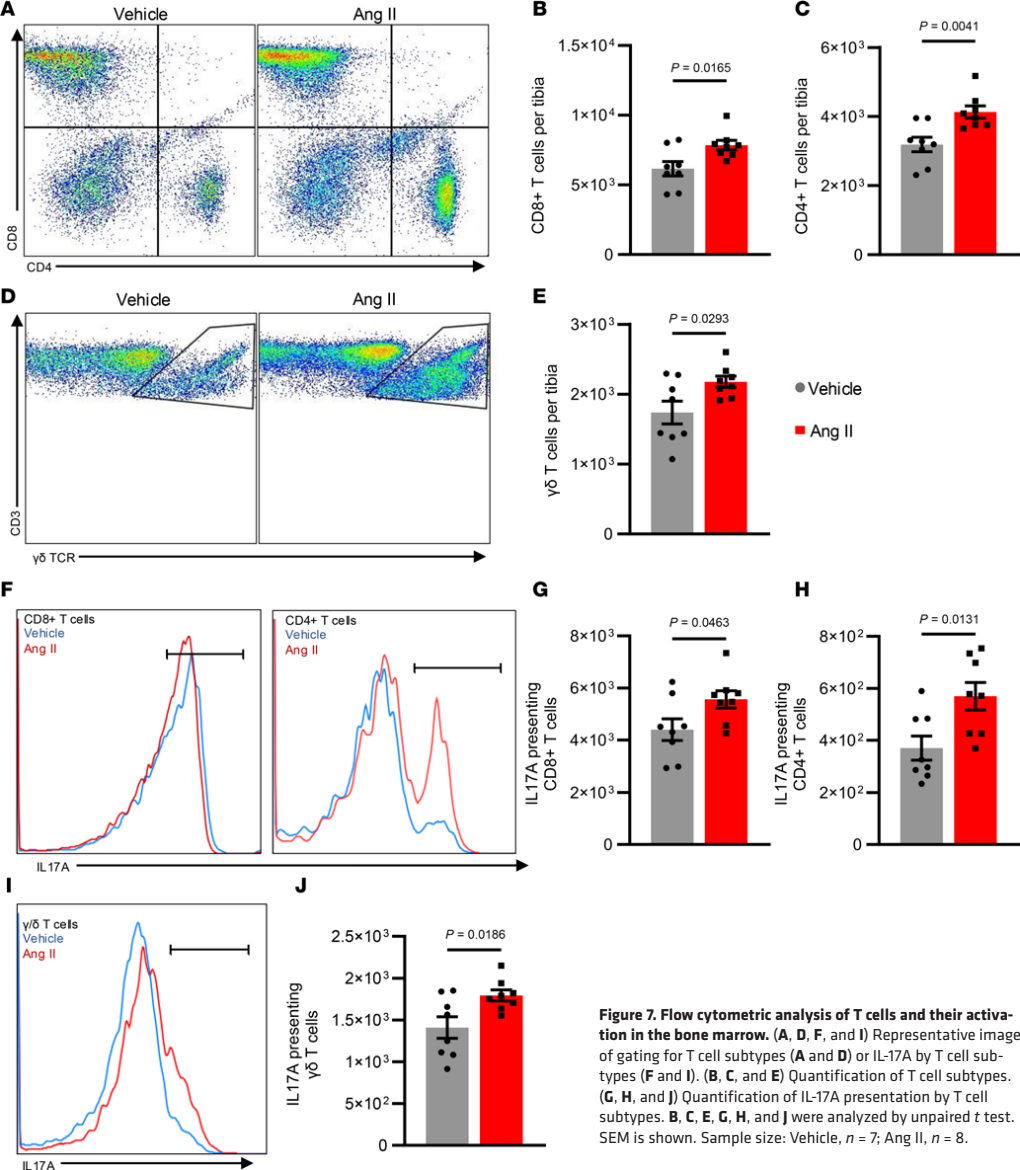
Flow cytometric analysis of T cells and their activation in the bone marrow. (**A**, **D**, **F**, and **I**) Representative images of gating for T cell subtypes (**A** and **D**) or IL-17A by T cell subtypes (**F** and **I**). (**B**, **C**, and **E**) Quantification of T cell subtypes. (**G**, **H**, and **J**) Quantification of IL-17A presentation by T cell subtypes. **B**, **C**, **E**, **G**, **H**, and **J** were analyzed by unpaired *t* test. SEM is shown. Sample size: Vehicle, *n* = 7; Ang II, *n* = 8.

T cells and T cell–derived cytokines contribute to both hypertension (29) and osteoporosis (30). Therefore, we quantified T cell subtypes in the bone marrow of vehicle- and Ang II–infused mice using flow cytometry. Ang II–infused mice had significantly more CD8^+^, CD4^+^, and γ/δ T cells compared with vehicle-infused mice (Figure 7, A–E). Additionally, IL-17A is an integral cytokine in the pathogenesis of both hypertension and osteoporosis (31, 32). Ang II–infused mice had significantly more IL-17A–producing CD8^+^, CD4^+^, and γ/δ T cells than observed in vehicle-infused mice (Figure 7, F–J). We observed increased CD8^+^, CD4^+^, and γ/δ T cells in the bone marrow in mice with DOCA-salt hypertension compared with controls (Supplemental Figure 6, A–E). IL-17A presentation by CD8^+^ and γ/δ T cells significantly increased while it only moderately increased in CD4^+^ T cells during DOCA-salt hypertension (Supplemental Figure 6, F–J).

**Figure 8:**
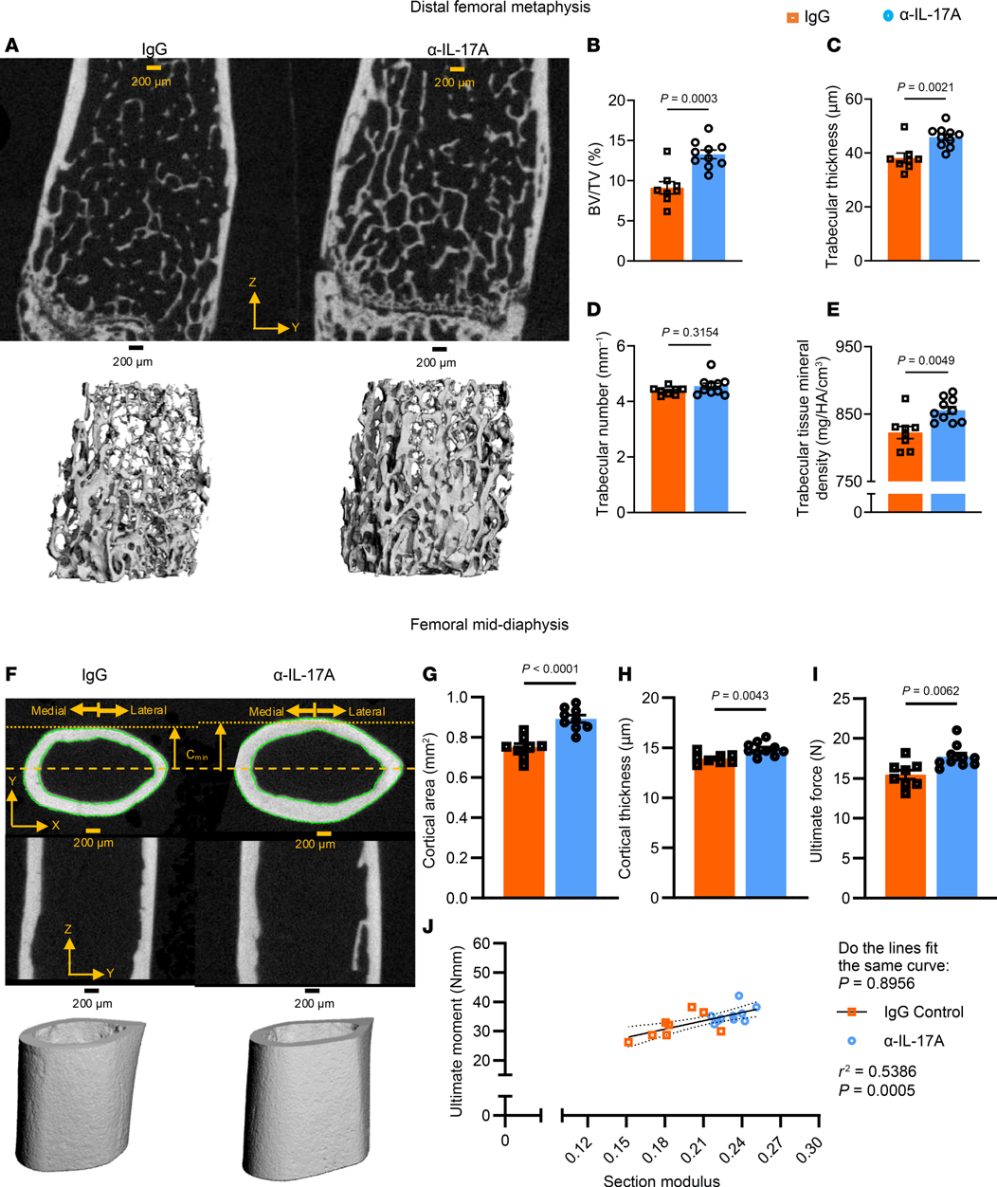
Trabecular architecture of the distal femoral metaphysis and cortical structure of the femoral mid-diaphysis from Ang II–infused mice treated with IgG or α-IL–17A. (**A**) Representative images of 2D (top) and 3D (bottom) renderings of the metaphysis. (**B**–**F**) Quantification of trabecular BV/TV (**B**), thickness (**C**), number (**D**), and tissue mineral density (**E**). (**F**) Representative images of 2D (top) and 3D (bottom) rendering of the diaphysis. (**G** and **H**) Cortical area (**G**) and thickness (**H**). (**I**) Ultimate force. (**J**) Correlation of section modulus and ultimate moment for IgG-treated (orange) and α-IL-17A–treated (blue) mice. **B**, **C**, **E**, and **G**–**I** were analyzed by unpaired *t* test. **D** was analyzed by Mann-Whitney test. **J** was analyzed by nonlinear regression. SEM is shown. Sample size: IgG, *n* = 8; α-IL-17A, *n* = 10. Scale bars: 200 μm.

To understand the pathogenic role of IL-17A in hypertension-induced bone loss, mice infused with Ang II were given either anti–IL-17A (α-IL-17A) or IgG isotype control by intraperitoneal injections every other day for 6 weeks. Saleh et al. previously showed that immunoclearing or genetic deletion of IL-17A markedly blunts Ang II–induced hypertension, using radiotelemetry (10, 11). We confirmed this in the present study using noninvasive tail-cuff measurements of SBP (Supplemental Figure 7A). Heart rate was not changed by IL-17A immunoclearing (Supplemental Figure 7B). The distal femoral metaphysis of the α-IL-17A–treated mice had significantly higher BV/TV relative to IgG-treated mice (Figure 8, A and B). There was an increase in trabecular thickness (Figure 8C) but no change in trabecular number (Figure 8D) or separation (Supplemental Table 5). Tissue mineral density of trabecular bone was preserved in α-IL-17A–treated mice (Figure 8E). There were significant differences in structure index (Supplemental Table 5). The α-IL-17A–treated mice also exhibited increased cortical area and thickness (Figure 8, F–H). α-IL-17A–treated mice had reduced cortical porosity compared with IgG-treated hypertensive mice (Supplemental Table 5). These changes in bone mass paralleled the increase in ultimate force withstood by femora from α-IL-17A–treated mice (Figure 8I). This is likely explained by the increased total area, minimum moment of inertia, and polar moment of inertia (Supplemental Table 5). α-IL-17A–treated mice also had increased anterior-posterior diameter and femur length (Supplemental Table 5). These structural parameters were also reflected by the distribution of these 2 groups on the best-fit line correlating section modulus and ultimate moment (Figure 8J).

We also examined the bone mass and strength of L6 VB from IgG- and α-IL-17A–treated mice. As in the distal femoral metaphysis, there was a significant increase in the BV/TV in the L6 VB of α-IL-17A–treated mice compared with IgG-treated mice (Supplemental Figure 8, A and B). This corresponded with a marked increase in trabecular thickness (Supplemental Figure 8C); however, there was no change in trabecular number (Supplemental Figure 8D) or separation (Supplemental Table 6). Tissue mineral density significantly increased with α-IL-17A treatment (Supplemental Figure 8E). α-IL-17A–treated mice had an increase in trabecular connectivity density and cross-sectional bone area, while the structure index decreased (Supplemental Table 6). Treatment with α-IL-17A significantly increased estimated failure load as quantified by μFEA (Supplemental Figure 8F) but not ultimate force by compression testing (Supplemental Figure 8G) in comparison with IgG-treated mice.

**Figure 9:**
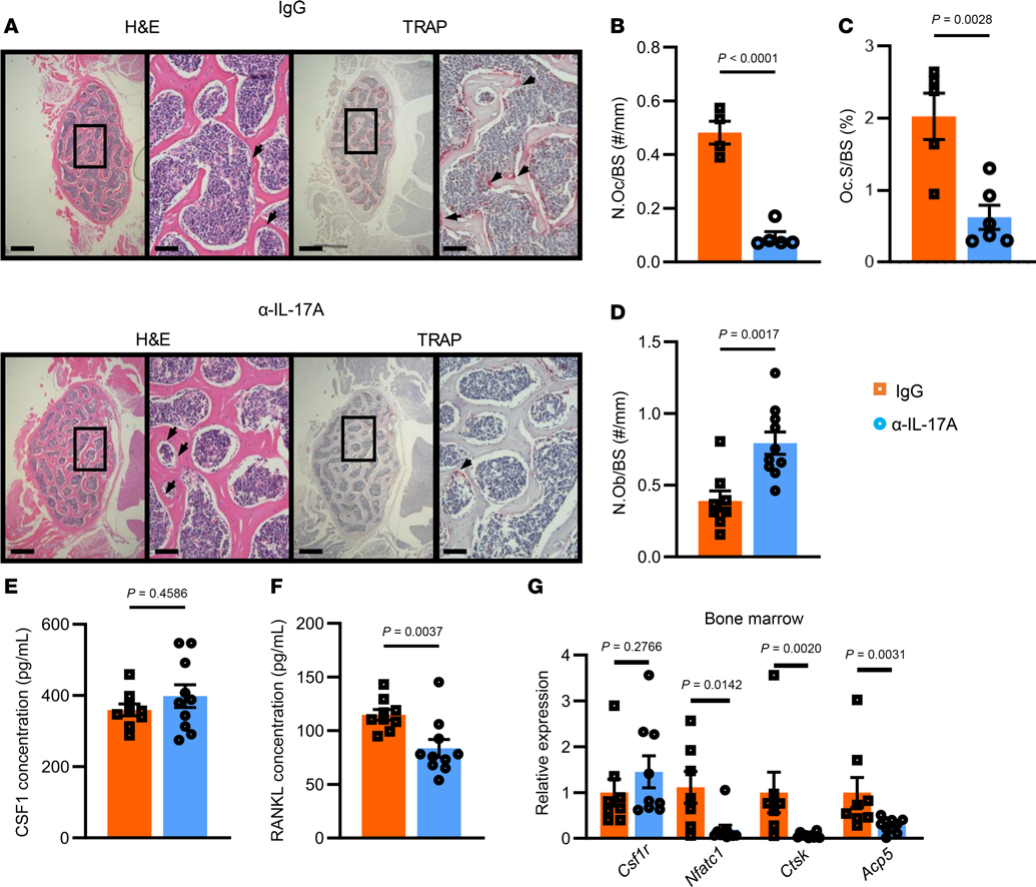
Analysis of osteoclasts in the bone and bone marrow from Ang II–infused IgG- or α-IL-17A–treated mice. (**A**) H&E-stained and TRAP-stained sections at 2 views (original magnification, ×4 at left and ×20 at right). Left scale bars: 200 μm; right scale bars: 50 μm. (**B**–**D**) Quantification of differences in osteoclast number (**B**) or surface (**C**) and osteoblast number (**D**). (**E**) The concentration of CSF1 in the bone marrow. (**F**) The concentration of RANKL in the bone marrow. (**G**) The relative expression of osteoclast-related transcripts. **B**–**E** were analyzed by unpaired *t* test. **F** and **G** were analyzed by Mann-Whitney test. SEM is shown. Sample size: IgG, *n* = 5–7; α-IL-17A, *n* = 6–10.

We also examined the effect of α-IL-17A treatment on osteoclasts and osteoblasts. Treatment with α-IL-17A significantly reduced N.Oc/BS and Oc.S/BS and increased N.Ob/BS compared with IgG treatment (Figure 9, A–D). In the bone marrow supernatant, α-IL-17A–treated mice had a significant decrease in RANKL concentration with no changes in CSF1 concentration (Figure 9, E and F). RNA was extracted from the bone marrow to measure transcript levels of osteoclast markers, and cells from α-IL-17A–treated mice exhibited significant reductions in *Ctsk*, *Acp5* (TRAP), and *Nfatc1* mRNA expression, while there were no changes in *Csf1r* expression (Figure 9G). There were no significant differences in the number of monocytes and macrophages in the bone marrow as assessed by flow cytometry (Supplemental Figure 9, A–D).

We also measured P1NP, CTX-1, and TRAP in the serum of IgG- and α-IL-17A–treated mice. α-IL-17A mice had a significant increase in P1NP and a significant decrease in CTX-1 and TRAP (Supplemental Figure 10, A–C). This implies that osteoclastic activity decreased while osteoblastic activity increased in response to immunoclearing of IL-17A. IgG-treated mice exhibited no significant correlation between P1NP and CTX-1, while α-IL-17A–treated mice exhibited a positive correlation between these two (Supplemental Figure 10D), suggesting that immunoclearing of IL-17A may recover the coupling of osteoblasts and osteoclasts.

### CSF1/CSF1-R signaling in hypertensive mice.

**Figure 10:**
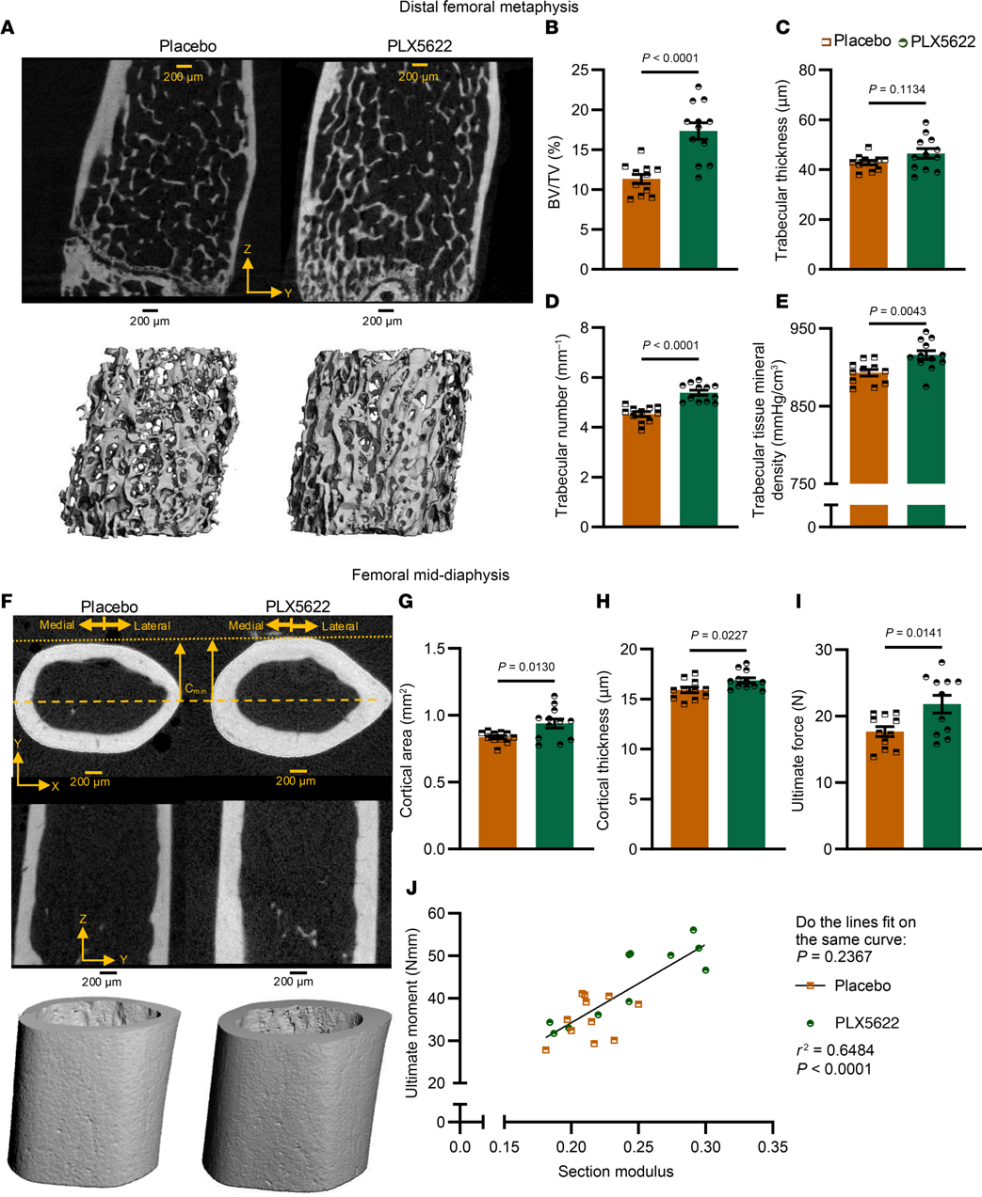
Trabecular architecture of the distal femoral metaphysis and cortical structure of the femoral mid-diaphysis from Ang II–infused mice treated with placebo or PLX5622. (**A**) Representative images of μCT 2D (top) and 3D (bottom) renderings of the distal femoral metaphysis. (**B**–**F**) Quantification of trabecular BV/TV (**B**), thickness (**C**), separation (**D**), and tissue mineral density (**E**). (**F**) Representative images of μCT 2D (top) and 3D (bottom) rendering of the femoral mid-diaphysis. (**G** and **H**) Quantification of cortical area (**G**) and thickness (**H**). (**I**) Ultimate force. (**J**) The correlation between section modulus and ultimate moment for placebo (orange) and PLX5622 (green) mice. **B**–**E**, **G**, and **H** were analyzed by unpaired *t* test. **H** was analyzed by Mann-Whitney test. **J** was analyzed by nonlinear regression. SEM is shown. Sample size: placebo, *n* = 11; PLX5622, *n* = 12. Scale bars: 200 μm.

CSF1 is an important driver of macrophage and osteoclast differentiation. Previous work by De Ciuceis et al. showed that CSF1-deficient mice (Op/Op mice) are protected against hypertension and exhibit reduced macrophage populations (13). To study the role of CSF1 in bone loss in hypertension, we treated mice with an inhibitor of CSF1 receptor (CSF1-R) (PLX5622, MedChem Express) in their chow, beginning 1 week before Ang II infusion. The hypertensive response to Ang II was not changed by PLX5622 treatment (Supplemental Table 7). In contrast, the distal femoral metaphysis of PLX5622-treated mice had significantly higher trabecular BV/TV (Figure 10, A and B) due to an increased number but not thickness (Figure 10, C and D). PLX5622 also increased tissue mineral density (Figure 10E) in comparison with placebo-treated mice. PLX5622 decreased trabecular separation and structure index while increasing connectivity density (Supplemental Table 8). Inhibition of CSF1-R also increased cortical area and thickness (Figure 10, F–H) in the mid-diaphysis in comparison with placebo-treated mice. PLX5622 decreased cortical porosity and increased anterior-posterior diameter while not changing other parameters (Supplemental Table 8). PLX5622 treatment resulted in a significantly higher ultimate force (Figure 10I). PLX5622 did not affect the best-fit line correlating section modulus and ultimate moment (Figure 10J).

Like the distal femoral metaphysis, PLX5622 exhibited protective effects on the trabecular architecture of the L6 VB (Supplemental Table 9). PLX5622-treated mice had significantly increased trabecular BV/TV (Supplemental Figure 11, A and B) compared with placebo-treated hypertensive mice. While PLX5622 did not affect vertebral trabecular thickness (Supplemental Figure 11C), it significantly prevented a decrease in trabecular number (Supplemental Figure 11D). PLX5622 did not affect tissue mineral density (Supplemental Figure 11E). PLX5622 treatment also prevented the deleterious effects of Ang II–induced hypertension on estimated failure load and ultimate force (Supplemental Figure 11, F and G).

In keeping with these beneficial effects of CSF1-R inhibition, PLX5622 increased P1NP and decreased CTX-1 and TRAP in the hypertensive mice compared with placebo-treated mice (Supplemental Figure 12, A–C). There was no obvious correlation between P1NP and CTX-1 following CSF1-R inhibition (Supplemental Figure 12D). We also examined the effect of CSF1-R inhibition on the bone marrow mRNA transcriptome using NanoString analysis. Significantly upregulated genes included *PPARgc1B* (peroxisome proliferator–activated receptor γ, coactivator 1β), *CSF1*, and *KLF4* (Kruppel-like factor 4), among others (Supplemental Figure 12E and Supplemental Table 10). Kyoto Encyclopedia of Genes and Genomes (KEGG) analysis of the significantly different genes suggests enrichment for pathways including osteoclast differentiation and pathways regulating pluripotency of stem cells, proteoglycans, cytokine–cytokine receptor interaction, calcium signaling, and PI3K/Akt (phosphatidylinositol 3-kinase/protein kinase B) signaling (Supplemental Figure 12F). Flow cytometric analysis of the bone marrow from these mice showed that inhibitor-treated hypertensive mice had a marked reduction in monocytes and macrophages (Supplemental Figure 12, G–J).

### Markers of bone turnover in humans with hypertension.

**Figure 11:**
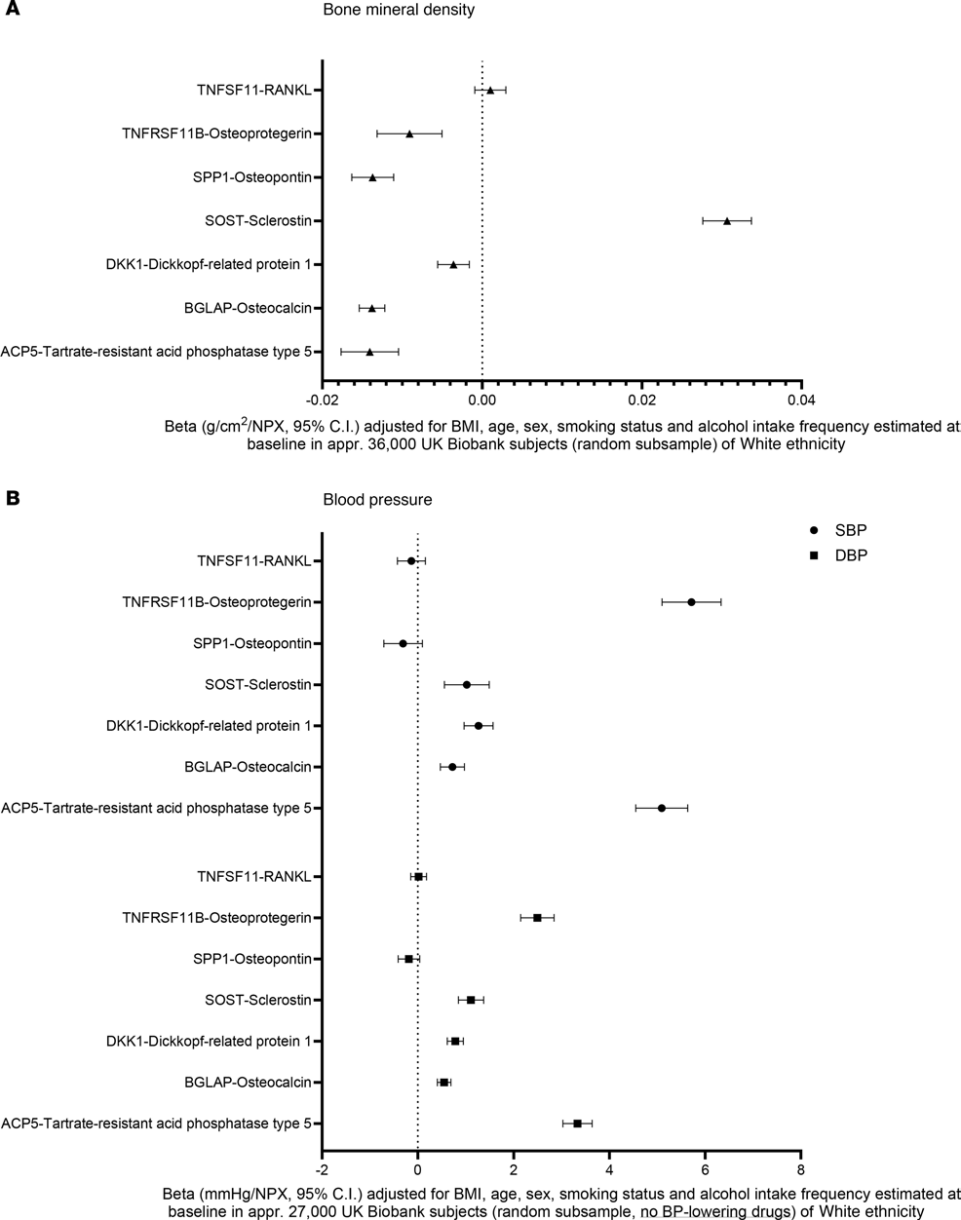
Forest plot of areal BMD or blood pressure versus normalized protein concentration (NPX) of bone remodeling biomarkers from patients of White ethnicity in the UK Biobank. (**A**) Beta values (g/cm^2^/NPX, 95% CI) comparing areal BMD versus NPX. (**B**) Beta values (mmHg/NPX, 95% CI) for SBP and DBP. Significance is denoted by the confidence interval not overlapping with the vertical dotted line. Data were adjusted for BMI, age, sex, smoking status, and alcohol intake frequency estimated at baseline. **A** consists of approximately 36,000 UK Biobank subjects (random subsample), while **B** sampled 27,000 subjects not on blood pressure–lowering drugs.

Our experimental data indicated that hypertension enhances osteoclast activity in 2 models of hypertension. To determine the relationship between markers of bone metabolism and blood pressure in humans, we leveraged the availability of Olink biomarker data from a random sample of approximately 36,000 White UK Biobank subjects (mean age 56.9 ± 8.0 years, mean body mass index 27.4 ± 4.7 kg/m^2^, 46% males, mean SBP 138.0 ± 18.5 mmHg, mean BMD 0.54 ± 0.12 g/cm^2^) or 27,000 UK Biobank subjects not receiving blood pressure medication. The former dataset was used to determine significant correlations between known bone metabolism markers and areal BMD. We observed a significant association between decreasing areal BMD and OPG, osteopontin, Dickkopf1 (DKK1), osteocalcin, and TRAP (Figure 11A). Increasing areal BMD is associated with Sost (Figure 11A). These same markers were correlated with changes in SBP and diastolic blood pressure (DBP). We observed a striking association between OPG and TRAP in both SBP and DBP, such that there was a 6 mmHg increase in SBP per arbitrary normalized protein concentration (NPX) unit increase in OPG and a 5.5 mmHg increase in systolic pressure per NPX unit increase in TRAP (Figure 11B). Changes in blood pressure were not associated with changes in RANKL (Figure 11B). OPG and TRAP levels were similarly associated with diastolic pressures (Figure 11B). Moreover, increases in SOST, DKK1, and osteocalcin were also associated with increases in SBP and DBP (Figure 11B). Hypertensive mice from either model similarly had increased circulating OPG and SOST (Supplemental Figure 13), which may suggest a mechanistic link between these proteins and hypertension-induced bone loss.

## Discussion

While humans with hypertension are more likely to experience a fracture than normotensive age- and sex-matched adults, the mechanism by which this disease weakens bone is unknown. In the present study, hypertensive mice exhibited significant declines in bone mass and strength in two distinct preclinical models of hypertension. In both models, we observed increased IL-17A–producing T cells. Immunoclearing of IL-17A during Ang II–induced hypertension led to preservation of bone mass and strength and reduced IL-17A–mediated osteoclastogenesis. Moreover, hypertension increased bone marrow monocytes and macrophages, which are likely precursors to osteoclast formation; however, some osteoclasts are yolk sac derived (27). We found this was associated with transcriptomic changes in the bone and bone marrow, supporting osteoclastogenesis and osteoclast activity. We further show that the cytokine CSF1 likely contributes to bone loss, as CSF1 transcripts and marrow CSF1 protein levels are increased in hypertension, and that blocking CSF1-R ameliorated bone loss in Ang II–induced hypertension.

The two models of hypertension employed in this study have different pathophysiology, but largely similar effects on bone structure, architecture, and strength. The Ang II infusion model of hypertension mimics hypertension in humans with an activated renin-angiotensin system, often associated with high or normal renin levels, and normal or contracted plasma volumes (33). In contrast, the DOCA-salt model of hypertension markedly suppresses renin levels and is dependent on salt and volume retention (11). Despite these differences in pathophysiology, there are known similarities in the two forms of hypertension. Both are associated with abnormalities of vascular function (34–36), increases in sympathetic outflow (37, 38), and immune cell activation (11). In both models, the blood pressure elevation was reduced in mice lacking T cells (11), by blocking of T cell activation (39, 40), or by blocking of inflammatory cytokines including IL-17A and IFN-γ (10, 41). One pathogenic difference in the models, however, is the reduced renal function, due to the nephrectomy necessary to promote hypertension, in the DOCA-salt model. We also found that the DOCA-salt model is associated with a modest increase in circulating PTH. These factors may contribute to reductions in bound water and fracture toughness (Supplemental Figure 2). Also, the uncoupling of bone remodeling in the Ang II model (Supplemental Figure 5G) did not occur in the DOCA-salt model (Supplemental Figure 5H). The expression of osteoclast genes (*Tnfsf11* and *Ctsk* in Supplemental Figure 3E) and osteoblast genes (*Alpl* and *Col1a1* in Supplemental Figure 3H) was higher in DOCA-salt mice than in control mice, whereas osteoclast gene expression (Supplemental Figure 3B) was only higher in Ang II–infused mice with no difference in osteoblast gene expression (Supplemental Figure 3G) between Ang II– and vehicle-infused mice.

A striking finding in the current study was that hypertension causes marked changes in expression of pro-osteoclastic mRNA transcripts in the bone and bone marrow. These included *Tnfsf11*, *Csf1*, *Ctsk*, *Nfatc1*, and *Acp5*. While hypertension is known to have effects on the bone marrow niche (42, 43), the finding that similar transcript-level changes are observed in the bone itself is new and suggests that bone is a previously unrecognized end organ in hypertension. It is likely that factors such as increased sympathetic tone, oxidative injury, alterations of bone marrow endothelial cells, and potential alterations in mechanical forces affect bone marrow function. As examples, increased sympathetic tone has been shown to promote bone loss (44), and oxidative injury promotes osteoclast formation (45). These events can affect the adjacent bone matrix. In this way, the bone marrow and bone can act as both initiators and end organs in hypertension.

The finding that IL-17A–producing cells are increased in hypertension is in keeping with prior observations that IL-17A–deficient mice are protected from hypertension, vascular dysfunction, and vascular fibrosis induced by Ang II infusion (31). Likewise, immunoclearing of IL-17A or immunoblockade of the IL-17A receptor prevents experimental hypertension (10, 11). IL-17A has been shown to signal inhibitory phosphorylation of endothelial cell nitric oxide synthase via activation of Rho kinase (32), and to promote renal sodium resorption (8). Similarly, IL-17A levels are increased in humans with hyperparathyroidism and mediate bone loss in response to chronic parathyroid hormone infusion (46). This latter effect is dependent on the ability of IL-17A to stimulate osteocytic RANKL production (16).

Our study shows that untreated-hypertensive individuals within the UK Biobank have significantly upregulated circulating biochemical markers for bone remodeling relative to their SBP or DBP. Increased SOST and DKK1, which inhibit bone formation, and reduced osteocalcin suggest that less bone formation occurs in hypertensive individuals. Increased TRAP suggests that there are increased osteoclast differentiation and activity in hypertensive adults. These biochemical markers have all been clinically associated with changes in bone metabolism, osteoporosis, and fracture (47–50). Taken together, an increase in blood pressure appears to be associated with less bone formation and more resorption despite increases in circulating OPG with no changes in RANKL. This is compatible with the concept that blood pressure and bone turnover are closely linked in humans. Interestingly, these markers also play a role in cardiovascular disease. For example, DKK1 is associated with cardiovascular disease in patients with type 2 diabetes (51), and TRAP is associated with coronary atherosclerosis and artery disease (52, 53). OPG is generally associated with an inhibition of osteoclastogenesis, and its increase could represent a compensatory mechanism in hypertensive individuals. OPG has also been implicated in the pathophysiology of chronic kidney disease (54) and is associated with left ventricular hypertrophy and systolic dysfunction (55). Potentially, these circulating markers are differentially regulated during hypertension because of the dysregulation of bone metabolism. These biomarkers may be useful to identify patients at risk for hypertension-induced bone loss, allowing for prophylactic care to mitigate long-term risk for osteoporotic fractures.

This study specifically identifies IL-17A as an integral component of bone loss during hypertension; however, other cytokines, like TNF-α, IL-6, and IL-23, work concurrently with IL-17A or increase its production and are implicated in osteoclast formation and activation (56–58). Additionally, this study shows an increase in monocytes and macrophages that is independent of IL-17A. CSF1/CSF1-R signaling is essential for hematopoietic stem cell commitment, myelopoiesis, and pre-osteoclast differentiation (59). We show that inhibiting CSF1-R blunted hypertension-induced loss in bone mass and strength (Figure 10 and Supplemental Figure 12). Together, these data support the concept that inflammatory activation during hypertension promotes loss in bone mass and strength.

One limitation of this study is the use of only male mice. Notably, both osteoporosis and hypertension exhibit sexual dimorphisms such that premenopausal women are protected against hypertension and osteoporosis, but the incidence of these conditions increases exponentially after menopause (60, 61). The present study also did not establish the origins of the osteoclasts in hypertension as being predominantly from bone marrow monocytes or from tissue-resident macrophages. Regardless, hypertension appears to favor osteoclastogenesis. Studies of human bone samples from age- and sex-matched patients with and without hypertension could also delineate the relative contributions of age and hypertension to bone fragility but would require large numbers of subjects. Our analysis of the UK Biobank clearly implicates an interaction of hypertension with bone loss in humans. Lastly, since bone mass decreases with age without elevated blood pressure, it remains to be determined whether hypertension causes a similar loss or differential loss in bone strength between young and aged animals.

In summary, this study emphasizes that the bone is a previously unrecognized mediator and target of hypertension. We show that there is a striking effect of hypertension on the makeup of bone marrow cell populations and bone biomarkers, in keeping with the concept that the bone marrow is an initiator of hypertension (62). In addition, we show that bone is a target of end-organ damage in hypertension, similarly to the vasculature, the kidney, and the brain. Clinical management of hypertension should entail evaluation of bone strength and density, particularly in patients at higher risk for osteoporosis.

## Methods

### Sex as a biological variable.

Male mice were used because estrogen protects female mice from experimental hypertension. Human data came from a large cohort of subjects that enabled comparisons to be adjusted for sex.

### Animals.

Male C57BL/6J mice (catalog 000664, The Jackson Laboratory) at 12 weeks of age were randomly selected from their cages and assigned to groups within 2 models of hypertension. For Ang II induction of hypertension, mice received a subcutaneous infusion of Ang II (490 ng/kg/min × 6 weeks) via osmotic minipumps (catalog 2006, Alzet). Vehicle-treated animals received osmotic minipumps containing only Ang II diluent. For DOCA-salt hypertension, mice underwent a right nephrectomy by flank incision, a DOCA (100 mg, catalog M-121, Innovative Research of America) pellet was implanted subcutaneously, and 1% NaCl was added to the drinking water for the following 3 weeks. Control mice underwent uninephrectomy but received a control pellet (catalog C-111, Innovative Research of America) and were not provided with NaCl in their drinking water.

In some experiments, mice undergoing Ang II infusion were given 100 μg/kg monoclonal antibody against IL-17A (catalog 16-7173085, eBioscience) or its IgG isotype control (Mouse IgG1 kappa P3.6.8.1, catalog 16-4714-85, eBioscience). Intraperitoneal injections began 2 days before the implantation of Ang II osmotic minipumps and were administered every other day until sacrifice. In other experiments, mice undergoing Ang II infusion were fed either a control synthetic diet (D11112201, Research Diets) or the same diet containing PLX5622, a small-molecular inhibitor of CSF1-R (MedChemExpress). The diet was given 1 week before osmotic minipump surgery and continued for the total infusion period.

At the time points indicated, mice were euthanized by CO_2_ inhalation before tissue collection. All procedures were approved by the Vanderbilt University Institutional Animal Care and Use Committee and were conducted in an Association for Assessment and Accreditation of Laboratory Animal Care International–accredited facility in accordance with the *Guide for the Care and Use of Laboratory Animals* and the US Public Health Service Policy on Humane Care and Use of Laboratory Animals.

### Blood pressure measurement.

Blood pressure was measured using a noninvasive tail-cuff system (MC4000 Blood Pressure Analysis System, Model MC4000MSP, Hatteras Instruments). All measurements were done 1 week before sacrifice. Mice given antibodies for IL-17A or isotype control had baseline blood pressure measurements taken 1 week before implantation. Mice were acclimatized to the tail-cuff procedure for 2 consecutive days before blood pressure measurement. All measurements were conducted from 9 to 11 am over a 2-day period. The mice acclimated to the machine for 5 minutes before measurements to reduce stress and increase body temperature for more accurate results. A minimum of 20 blood pressure readings were averaged from each animal to obtain final readings of SBP and heart rate.

### Transdermal measurement of glomerular filtration rate.

One week before sacrifice, mice received FITC-sinistrin by retro-orbital injection, and transdermal glomerular filtration rates (tGFRs) were measured in conscious mice, as previously described (25). The FITC-sinistrin (0.15 mg/g; catalog NC1570801, MediBeacon) half-life was calculated using a 3-compartment model with linear fit using MPD Studio software (MediBeacon, Mannheim, Germany), and the FITC-sinistrin half-life was converted to tGFR (in mL/min) with correction for mouse body weight.

### Micro–computed tomography analysis.

Micro–computed tomography (μCT) analyses were performed using a Scanco μCT 50 system (Scanco Medical AG). After securing of each femur in a μCT specimen tube holder (part U50821, Scanco Medical AG), the mid-diaphysis and distal femoral metaphysis were scanned. Each femur was scanned at an isotropic voxel size of 6 μm (70 kVp, 114 μA, 8 W; 0.1 mm aluminum filter, 1,024 samples per 1,000 projections per 360° rotation; and 300 milliseconds integration time at 2 frame averages) in PBS. A manufacturer-recommended beam hardening correction factor (BH: 1,200 mg hydroxyapatite [HA]) was used during image acquisition to reduce any signal artifacts. The HA phantom calibration was used to convert the x-ray attenuation to a known density value. After the raw image stack was reconstructed, the scans of the femur mid-diaphysis and distal femur metaphysis were evaluated as described previously (63).

VBs from the same mice were scanned at an isotropic voxel size of 12 μm (55 kVp, 200 μA, 11 W; 0.5 mm aluminum filter; BH: 1,200 mg HA/cm^3^; 852 samples per 1,000 projections per 360° rotation; and 600 milliseconds integration time at 2 frame averages) in PBS. The cranial-caudal axis of the VB was aligned with the *z* axis of the specimen tube holder for the scanner. Evaluations of the VBs were done as described previously (64).

### μCT finite element analysis.

Before mechanical testing, the VBs were modeled and assessed using finite element analysis. To specify the volume of interest used to create 3D reconstructions of the vertebrae, a circle with a constant radius of 1.24 mm was copied into each image between the end plates and positioned to transect the transverse processes, which did not bear load in the compression as previously described (65). Vertebral end plates were not included in the model. Image noise was reduced using a Gaussian filter with a sigma of 0.2 and support of 1. Scanco FE software (fe_solve3, v1.13, Scanco Medical AG) was used to convert each voxel into 8-node brick elements. The strain experienced by each element was calculated for simulated high-friction, axial compression loading of VBs to a peak level of 1% apparent strain. Caudal nodes were constrained in the *x*-, *y*-, and *z*- directions, while the cranial nodes were constrained in the *x* and *y* directions. The cranial nodes had a defined negative displacement in the *z* direction. The base model elements were given a homogeneous elastic modulus (18 GPa) and Poisson’s ratio (0.3) (65). The reaction force at failure was reached when 2% of the model reached an equivalent strain of 0.007 as established previously (65).

### Assessment of bone biomechanics.

Bone biomechanical testing was performed using a material-loading system (Dynamight 8841, Instron). For assessment of long bone biomechanics, intact left femora were placed in a 3-point bending configuration with the anterior side down and medial side forward. With the span between the lower supports set to 8 mm, the hydrated bones were loaded at a rate of 3.0 mm/min until failure. Analysis was performed as described previously (63).

In the contralateral right femora, micronotching was performed to assess fracture toughness. The posterior side of the femora was machined into single-edge notched beam (SENB) specimens. The crack path region was scanned by the Scanco μCT 50 system (Scanco Medical AG) with an isotropic voxel size of 6 μm, and the corresponding volumetric BMD was determined. The SENB specimens were subjected to 3-point bending at a loading rate of 0.5 mm/min as previously described (63).

Compression testing was performed to assess vertebral biomechanics. After removal of the end plates and trimming of the transverse processes, a flat cylindrical platen (2.0 mm diameter) was used to compress the L6 VB to failure at a rate of 3 mm/min. From the resulting force-displacement curve, we determined the biomechanical properties as follows: stiffness (N/mm) was the slope of the linear portion of the curve, yield force (N) occurred when the slope of the aforementioned curve reached a 0.2% slope offset from the linear portion of the curve, and peak force (N) was the maximum force endured by the VB. Further description has previously been provided (64). Mechanical properties were quantified using a Matlab (MathWorks) script.

### ^1^H-NMR measurements.

After thawing to room temperature, each right femur was blotted and weighed in air (wet mass) and weighed while submerged in water to determine the volume estimate of the bone. The hydrated femur was air-dried again for a minute and then sealed in a glass NMR tube (catalog 662001075, Wilmad Labglass). This tube was inserted into a custom low-proton, loop-gap-style radiofrequency (RF) coil. A microsphere of a known volume of water (21.2 μL) was used as a reference volume. Ten thousand echoes at 100-microsecond echo spacing were acquired for 90° RF pulses with a duration of 6 microseconds or 180° at 12 microseconds using a 4.7-T horizontal-bore magnet (Varian Medical Systems). The echo signal intensity was processed and analyzed as previously reported (63).

### Flow cytometry.

To quantify immune cells present in the bone marrow, the medullary canal of the right tibia was flushed with PBS. Samples were filtered through a 40 μm cell strainer and centrifuged at 350*g* for 7 minutes. The pellet was then resuspended in 1 mL of red blood cell lysis buffer (catalog 00-4333-57, Invitrogen) for 1 minute. The resultant single-cell suspensions were washed and stained for the indicated surface markers. Cells were then fixed with Fix and Perm Medium A (catalog GAS001, Invitrogen) and stained for the indicated intracellular markers in Fix and Perm Medium B (catalog GAS002, Invitrogen). Flow cytometry was performed using Cytek Aurora (Cytek), and data were analyzed using FlowJo (Tree Star Inc.) software. Dead cells were excluded using live/dead staining. Results were expressed as the number of cells per tibia. Antibodies used and their fluorophore conjugates are shown in Supplemental Table 11. The gating strategy used in the study is presented in Supplemental Figures 14 and 15.

### Serum collection.

Blood was collected from the left ventricle of the heart using a heparinized syringe. Serum was isolated by allowing blood to clot for 30 minutes at room temperature. Then the blood was centrifuged at 4°C at 1,500× *g* for 10 minutes. Serum was aliquoted for the desired assays. Enzyme-linked immunosorbent assays (ELISAs) for P1NP, CTX-1, and TRAcP5b (Immunodiagnostics) were done as described by the manufacturer’s instructions. Serum from some mice was used for circulating parathyroid hormone measurements (Immutopics Inc.) and for SOST and OPG measurements per the manufacturer’s instructions (R&D Systems).

### Total RNA isolation.

The left and right humeri were cut with a razor blade at the distal epiphyses, and the cut end was placed into the bottom of a 0.6 mL Eppendorf tube containing 50 μL of 1× PBS. This was similarly done for a subset of right femora. The marrow was flushed from the bones by centrifugation at 10,000× *g* for 2 minutes (66). The supernatant was stored for ELISAs (CSF1 and RANKL, R&D Systems), leaving a pellet of cells. The mRNAs from these cells were extracted and purified. The bone was flash-frozen in liquid nitrogen and stored at –80°C. Later, the frozen samples were pulverized for 2 minutes at a rate of 14 cycles per second using a freezer/mill (6770, SPEX SamplePrep LLC) filled with liquid nitrogen. Total RNA was extracted from the bone powder using TRIzol (Invitrogen) and further processed using the RNeasy MinElute Cleanup kit (catalog 74204, QIAGEN). Samples were diluted such that 400 ng of RNA were in 8 μL of nuclease-free water. Some of these samples were submitted for NanoString analysis as described in the next section. For quantitative PCR, cDNAs were synthesized following DNase I treatment using a high-capacity cDNA reverse transcription kit (Applied Biosystems). TaqMan probes (Thermo Fisher Scientific) were used to quantify amplification of target transcript (Supplemental Table 12). StudioQuant 3 (Thermo Fisher Scientific) was used for amplification. Data were normalized against the Ct (cycle threshold) values of β-actin to assess fold change (2^–ΔΔCt^).

### NanoString nCounter analysis.

Gene-specific mRNA counts from the bone and bone marrow total RNA samples were quantified using an automated NanoString nCounter Mx system (NanoString Technologies; RRID: SCR_021712) with a custom probe set of 625 musculoskeletal genes by the UCSF Core Center for Musculoskeletal Biology and Medicine Skeletal Biology and Biomechanics Core as previously reported (67) and were analyzed using nSolver Analysis Software (NanoString Technologies) and nCounter Advanced Analysis Software (NanoString Technologies). Counts were normalized using *Gapdh*, *Rpl19*, and *Ldha*. Significant fold differences were determined by unpaired *t* tests as previously described (68). Gene enrichment was analyzed using the ShinyGO 0.82 application (https://bioinformatics.sdstate.edu/go/; ref. 69).

### Static bone histology.

For histology, formalin-fixed VB samples were decalcified in 10% EDTA (pH 7.4) for 3 days, dehydrated in 70% ethanol, cleared, and embedded in paraffin. Five-micrometer transverse sections were cut on glass slides, deparaffinized, and subjected to H&E or TRAP staining (70). Histomorphometry measurements were performed using the Bioquant Analysis System.

### UK Biobank Study.

Over 500,000 participants (aged 40–69) were recruited from 22 assessment centers throughout the UK between 2006 and 2010, encompassing a range of diverse environments to ensure a mix of socioeconomic backgrounds and ethnicities and a balance between urban and rural settings (71). The study collected comprehensive information on the physical and genetic characteristics, including data obtained through surveys, physical assessments, biological sample testing, and long-term monitoring of health-related results (71). The UK Biobank received ethical approval from the North West Multi-Centre Research Ethics Committee (11/NW/03820). All participants gave written informed consent before enrollment in the study, which was conducted by the principles of the Declaration of Helsinki.

We used baseline (2006–2010) data from the random subsample of UK Biobank participants who underwent plasma proteomic measurements using the Olink platform (Olink Proteomics AB) (72). Associations of SBP and DBP with selected plasma Olink-quantified proteins were performed using approximately 27,000 participants of White ethnicity with 2 automated BP readings available and no intake of BP-lowering medications reported (UK Biobank field IDs 6177 and 6153). Associations of areal BMD (UK Biobank field IDs 3084, 3148, averaged 4105 and 4124, averaged 4140 and 4145) with selected plasma Olink-quantified proteins were performed using approximately 36,000 UK Biobank participants.

### Statistics.

The normal distribution assumption was evaluated using the Anderson-Darling normality test. Normally distributed data were compared between 2 groups using a Student’s 2-tailed unpaired *t* test. Non-normally distributed data were compared using the Mann-Whitney test. Significance was defined as *P* less than 0.05. The data are expressed as the mean with either the standard deviation (SD) or standard error of mean (SEM) as described. SBP and heart rate for IgG- and α-IL-17A–treated mice were analyzed by 2-way repeated-measures ANOVA with Tukey’s post hoc test. These statistics and graphs were made in GraphPad Prism. KEGG analysis was done and graphed as described by Ge et al. (73). Analyses of averaged, automated BP parameters and areal BMD in the UK Biobank were performed in SPSS (version 29.0) using the general linear model adjusted for BMI, age, sex, smoking status, and alcohol intake frequency.

### Study approval.

All animal procedures were approved by Vanderbilt University’s Institutional Animal Care and Use Committee. The mice were housed and cared for in accordance with the *Guide for the Care and Use of Laboratory Animals* (National Academies Press, 2011), US Department of Health and Human Services. This research was conducted using the UK Biobank resource under Application 93156.

### Data availability.

All data are available in the Supporting Data Values file.

## Author contributions

EMH, DGH, and JSN designed the study. EMH, SU, NDLV, W Chen, JK, RD, and LAV conducted experiments and acquired the data. EMH, SU, MS, RWJ, RD, and JSN analyzed the data. TJG, MS, and ML designed the UK Biobank study and analyzed the data. W Chang designed the NanoString panel and collected the data. EMH analyzed the NanoString data. DMP, MPDC, W Chang, RWJ, DGH, and JSN supervised the study. EMH, JSN, and DGH wrote the manuscript. All authors reviewed and revised the manuscript.

## Supplementary Material

Supplemental data

Supporting data values

## Version 1 08/19/2025

In-Press Preview

## Version 2 10/15/2025

Electronic publication
