# Supplementary material for: Hypertension promotes bone loss and fragility by favoring bone resorption in mouse models
Source: J Clin Invest. 2025 Aug 19;135(20):e184325. doi: 10.1172/JCI184325 (PMC12547992; doi:10.1172/JCI184325)
Supplement: Supplemental data [file jci-135-184325-s137.pdf]

Hypertension Promotes Bone Loss and Fragility by Favoring Bone Resorption in Mouse Models

Elizabeth M. Hennen[1], Sasidhar Uppuganti[2,3], Néstor de la Visitación[4], Wei Chen[4], Jaya Krishnan[4], Lawrence A. Vecchi III[4], David M. Patrick[2,4], Mateusz Siedlinski[5,6], Matteo Lemoli[5,7], Rachel Delgado[8], Mark P. de Caestecker[8], Wenhan Chang[9], Tomasz J. Guzik[5,6], Rachelle W. Johnson[4], David G. Harrison[4,^], and Jeffry S. Nyman[1,2,3,^]

From the [1]Department of Biomedical Engineering, Vanderbilt University, [2]Department of Veterans Affairs, Tennessee Valley Healthcare System, [3]Department of Orthopedic Surgery, Department of Medicine, Vanderbilt University Medical Center, [4]Division of Clinical Pharmacology, Department of Medicine, Vanderbilt University, Medical Center, [5]Centre for Cardiovascular Sciences, University of Edinburgh, UK, [6]Department of Internal and Agricultural Medicine and Omicron Medical Genomics Laboratory, Jagiellonian University, Collegium Medicum, Krakow, Poland, Department of Medicine, [7]Department of Clinical and Experimental Sciences, University of Brescia, Brescia, Italy, [8]Division of Nephrology and Hypertension, Department of Medicine, Vanderbilt University Medical Center, [9]San Francisco VA Medical Center, Department of Medicine, University of California, San Francisco

^Corresponding authors: David G. Harrison, MD
2200 Pierce Ave
Nashville, TN. 37232
email: david.g.harrison@vumc.org o: (615) 875-3049

Jeffry S. Nyman, PhD (https://orcid.org/0000-0001-7403-7605)
Medical Center East, South Tower, Suite 4200
Nashville, TN 37232
email: jeffry.s.nyman@vumc.org o: (615) 936-6296

Conflict of Interest: All authors agree to the content in the manuscript and contributed to the work being presented.

Supplemental Table 1: Blood pressure and heart rate measurements. Values are mean ± SD and comparisons were made using unpaired t tests.

Property	Unit	Vehicle (n=7)	Ang II (n=8)	P-value	Control (n=10)	DOCA (n=6)	P-value
Systolic Blood Pressure	mmHg	108.3 ± 5.678	168.6 ± 8.193	<0.0001	113.2 ± 5.473	155.8 ± 6.969	<0.0001
Heart Rate	BPM	573.6 ± 62.89	547.9 ± 81.61	0.5116	582.9 ± 38.93	598.2 ± 75.03	0.5969

Supplemental Table 2: Trabecular architecture, cortical structure, and cortical strength of the distal femoral metaphysis from both models of hypertension. P-values marked with * were calculated using unpaired t test and + were calculated using Mann-Whitney test. Standard deviation is provided.

Property	Unit	Vehicle (n=15)	Ang II (n=16)	P-value	Control (n=16)	DOCA (n=12)	P-value
**Trabecular Architecture Properties**							
Separation	µm	214.5 ± 20.08	224.2 ± 11.14	0.0273+	216.1 ± 13.74	240.6 ± 12.21	<0.0001+
Connectivity Density	mm^{-3}	236.6 ± 59.98	242.4 ± 55.69	0.7405+	301.6 ± 104.7	284.0 ± 57.74	0.8731+
Structure Index	0:plate; 3:rod; 4:sphere	1.886 ± 0.2740	1.966 ± 0.2230	0.3823*	2.065 ± 0.2371	2.275 ± 0.2035	0.0205*
**Cortical Properties**							
Tissue Mineral Density	mgHA $/cm^3$	1214 ± 13.20	1222 ± 13.82	0.1209*	1193 ± 37.55	1207 ± 35.24	0.2748+
Volumetric BMD	mgHA $/cm^3$	1175 ± 12.76	1179 ± 14.07	0.4378*	1155 ± 37.46	1155 ± 31.18	0.9531*
Porosity	%	4.223 ± 0.2845	4.499 ± 0.4558	0.0912+	4.434 ± 0.3435	5.172 ± 1.220	0.0974+
Total Area	mm^2	2.109 ± 0.1534	2.077 ± 0.2124	0.6391*	2.083 ± 0.2071	2.134 ± 0.1812	0.5015*
Minimum Moment of Inertia	mm^4	0.1507 ± 0.02322	0.1409 ± 0.02728	0.2921*	0.1507 ± 0.03461	0.1302 ± 0.02479	0.0941*
Polar Moment of Inertia	mm^4	0.4956 ± 0.06354	0.4447 ± 0.07636	0.0539*	0.4782 ± 0.09354	0.4048 ± 0.06836	0.0302*
Medullary Volume	mm^3	2.296 ± 0.2197	2.408 ± 0.3052	0.2537*	2.674 ± 0.6245	3.285 ± 0.5024	0.0100*
Length	mm	14.97 ± 0.2997	14.71 ± 0.3290	0.0285*	14.04 ± 1.248	13.91 ± 1.067	0.6231+
Anterior Posterior Diameter	mm	1.361 ± 0.09078	1.316 ± 0.08663	0.1678*	1.363 ± 0.08475	1.340 ± 0.08592	0.4838*
**3pt Bending Mechanical Properties**							
Stiffness	N/mm	112.5 ± 17.03	90.36 ± 17.05	0.0011*	99.03 ± 15.34	69.04 ± 10.84	<0.0001+
Yield Force	N	13.08 ± 2.210	10.33 ± 1.841	<0.0001+	12.21 ± 1.199	7.784 ± 1.111	<0.0001*
Post Yield Displacement	mm	0.9453 ± 0.2532	0.8834 ± 0.2659	0.6260+	0.9340 ± 0.3754	1.145 ± 0.4068	0.1672*
Work to Fracture	kJ/mm^3	7.861 ± 3.642	7.212 ± 4.511	0.2127+	12.57 ± 3.003	8.968 ± 1.979	0.0013*

Supplemental Table 3: Trabecular architecture and mechanical properties of the sixth lumbar vertebral body from both models of hypertension. P-values marked with * were calculated using unpaired t test and + were calculated using Mann-Whitney test. Standard deviation is provided.

Trabecular Architecture Properties							
Property	Unit	Vehicle (n=15)	Ang II (n=16)	P-value	Control (n=16)	DOCA (n=12)	P-value
Separation	µm	179.0 ± 12.07	186.8 ± 14.29	0.0319*	191.3 ± 15.09	220.9 ± 22.77	0.0003*
Connectivity Density	mm^{-3}	239.3 ± 22.93	222.3 ± 15.89	0.0239+	204.1 ± 26.22	178.1 ± 31.79	0.0255*
Structure Index	0:plate; 3:rod; 4:sphere	0.7391 ± 0.4830	1.097 ± 0.3360	0.0243*	1.103 ± 0.3514	1.809 ± 0.4318	<0.0001*
Bone Area	mm^2	0.7622 ± 0.09267	0.6506 ± 0.07453	0.0009*	0.7090 ± 0.1056	0.5392 ± 0.1115	0.0003*
**Compression Testing**							
Stiffness	N/mm	178.5 ± 77.84	114.6 ± 43.47	0.0072+	137.1 ± 62.40	96.39 ± 68.61	0.0530+
Yield Force	N	31.08 ± 6.973	25.21 ± 6.520	0.0131+	28.36 ± 7.500	16.32 ± 5.823	<0.0001*
Post Yield Displacement	mm	0.2207 ± 0.1450	0.2910 ± 0.1550	0.2031*	0.2362 ± 0.2090	0.3159 ± 0.2956	0.5293+
Axial Stress	MPa	44.07 ± 5.967	42.55 ± 4.576	0.4298*	42.64 ± 8.856	33.28 ± 4.972	0.0029*

Supplemental Table 4: Differentially expressed genes in the bone and bone marrow of hypertensive mice.

**Differentially Expressed Genes: Ang II vs. Veh (Baseline)**					
**Bone Marrow (n=5)**			**Bone (n=11-12)**		
**Gene**	**Log2(Fold Change)**	**P-value**	**Gene**	**Log2(Fold Change)**	**P-value**
Itga2	-0.56	0.000395	Sost	-1.63	0.00161
Kdr	-0.946	0.000403	Atxn1l	-0.616	0.00223
Follistatin	-1.73	0.00073	Ihh	-2.09	0.00318
Alox12	-0.588	0.000756	Gnai1	-0.679	0.00318
Itgb3	-0.462	0.000868	Gdf7	-1.99	0.00377
Smad5	-0.385	0.00105	Comp	-0.95	0.00453
Vwf	-0.696	0.00144	Wnt5a	-1.05	0.00462
Itga1	-0.371	0.00168	Sox4	-1.23	0.00488
Prgn	-0.57	0.00187	Ddr2	-0.624	0.00561
Pparg	-0.756	0.00196	Tgfbr2	-0.509	0.00577
**Differentially Expressed Genes: DOCA vs. Con (Baseline)**					
**Bone Marrow (n=6-9)**			**Bone (n=6-9)**		
Acta1	-1.86	0.000731	Ly6a	-3.95	0.0000385
Pdk4	-1.28	0.00183	Ndufa6	3.37	0.00158
Cox15	0.387	0.00588	Igfbp4	0.727	0.00251
Car3	-1.33	0.00608	Efnb1	2.41	0.00312
Mef2d	-0.554	0.00808	Hdac5	1.81	0.00341
Tnni2	-1.52	0.00811	Ndufs7	1.64	0.00793
Pparg	-0.663	0.0103	Serpinb2	-1.69	0.0119
Atp5g1	-0.43	0.0175	Sgca	0.543	0.0251
Ndufv3	0.591	0.0177	Fgf2	1.54	0.0252
Fut4	0.583	0.0178	Tnfsf11	-1.38	0.0272

Supplemental Table 5: Trabecular architecture, cortical structure, and cortical strength of the distal femoral metaphysis from IgG- and α-IL-17A-treated Ang II mice. P-values marked with * were calculated using unpaired t test and + were calculated using Mann-Whitney test. Standard deviation is provided.

**Trabecular Architecture Properties**				
Property	Unit	IgG (n=8)	α-IL-17A (n=10)	P-value
Separation	μm	194.0 ± 9.055	183.6 ± 15.06	0.1056*
Connectivity Density	mm^{-3}	212.3 ± 36.13	219.2 ± 42.05	0.7618+
Structure Index	0:plate; 3:rod; 4:sphere	2.250 ± 0.1864	1.764 ± 0.2118	0.0001*
**Cortical Properties**				
Tissue Mineral Density	mgHA $/cm^3$	1239 ± 15.71	1242 ± 10.14	0.3941+
Volumetric BMD	mgHA $/cm^3$	1194 ± 13.57	1200 ± 10.47	0.2632+
Porosity	%	4.905 ± 0.2594	4.343 ± 0.2631	0.0003*
Total Area	mm^2	1.869 ± 0.1389	2.179 ± 0.04819	<0.0001+
Minimum Moment of Inertia	mm^4	0.1172 ± 0.01579	0.1620 ± 0.01006	<0.0001*
Polar Moment of Inertia	mm^4	0.3986 ± 0.07107	0.5252 ± 0.03464	0.0006+
Medullary Volume	mm^3	2.073 ± 0.1509	2.379 ± 0.07867	<0.0001*
Length	mm	14.54 ± 0.1546	15.01 ± 0.2371	0.0002*
Anterior Posterior Diameter	mm	1.308 ± 0.05922	1.396 ± 0.03307	0.0010*
**3pt Bending Mechanical Properties**				
Stiffness	N/mm	96.45 ± 6.160	117.1 ± 15.71	0.0044+
Yield Force	N	11.68 ± 1.543	11.91 ± 2.164	0.8077*
Post Yield Displacement	mm	0.9829 ± 0.3841	0.8561 ± 0.3123	0.4598+
Work to Fracture	kJ/mm^3	11.23 ± 2.360	12.20 ± 3.307	0.4937*

Supplemental Table 6: Trabecular architecture and mechanical properties of the sixth lumbar vertebral body from IgG- and α-IL-17A-treated Ang II mice. P-values marked with * were calculated using unpaired t test and + were calculated using Mann-Whitney test. Standard deviation is provided.

**Trabecular Architecture Properties**				
Property	Unit	IgG (n=8)	α-IL-17A (n=10)	P-value
Separation	µm	194.0 ± 9.055	183.6 ± 15.06	0.1056*
Connectivity Density	mm^{-3}	222.3 ± 15.89	239.3 ± 22.93	0.0239+
Structure Index	0:plate; 3:rod; 4:sphere	1.510 ± 0.1380	1.033 ± 0.3331	0.0001*
Bone Area	mm^2	0.6116 ± 0.08415	0.7252 ± 0.08220	0.0108*
**Compression Testing**				
Stiffness	N/mm	178.5 ± 77.84	114.6 ± 43.47	0.0072+
Yield Force	N	31.08 ± 6.973	25.21 ± 6.520	0.0131+
Post Yield Displacement	mm	0.2207 ± 0.1450	0.2910 ± 0.1550	0.2031*
Axial Stress	MPa	44.07 ± 5.967	42.55 ± 4.576	0.4298*

Supplemental Table 7: Blood pressure and heart rate measurements for placebo- and PLX5622-treated, ang II-infused mice. Values are mean ± SD and comparisons were made using unpaired t tests.

Property	Unit	Placebo (n=6)	PLX5622 (n=6)	P-value
Systolic Blood Pressure	mmHg	162.4 ± 7.883	153.8 ± 12.37	0.1822[*]
Heart Rate	BPM	588.8 ± 83.97	521.5 ± 29.98	0.3939[+]

Supplemental Table 8: Trabecular architecture, cortical structure, and cortical strength of the distal femoral metaphysis from placebo- and PLX5622-treated, ang II-infused mice. P-values marked with * were calculated using unpaired t test and + were calculated using Mann-Whitney test. Standard deviation is provided.

**Trabecular Architecture Properties**				
Property	Unit	Placebo (n=11)	PLX5622 (n=12)	P-value
Separation	μm	221.6 ± 17.15	189.2 ± 10.30	<0.0001
Connectivity Density	mm^{-3}	183.2 ± 37.49	283.2 ± 45.87	<0.0001*
Structure Index	0:plate; 3:rod; 4:sphere	2.017 ± 0.2568	1.472 ± 0.2905	0.0001*
**Cortical Properties**				
Tissue Mineral Density	mgHA /cm^3	1239 ± 14.97	1234 ± 14.50	0.4512*
Volumetric BMD	mgHA /cm^3	1198 ± 17.70	1198 ± 14.48	0.9396*
Porosity	%	3.925 ± 0.6502	3.383 ± 0.5195	0.0377*
Total Area	mm^2	0.4554 ± 0.05332	0.5240 ± 0.1040	0.0908+
Minimum Moment of Inertia	mm^4	0.1429 ± 0.01744	0.1662 ± 0.03614	0.0792+
Polar Moment of Inertia	mm^4	0.4554 ± 0.05332	0.5240 ± 0.1040	0.0908+
Medullary Volume	mm^3	2.204 ± 0.2412	2.167 ± 0.2392	0.7177*
Length	mm	14.89 ± 0.3835	14.94 ± 0.3359	0.6391+
Anterior Posterior Diameter	mm	1.261 ± 0.04119	1.307 ± 0.07107	0.0172*
3pt Bending Mechanical Properties				
Stiffness	N/mm	107.9 ± 12.53	136.6 ± 31.13	0.0759+
Yield Force	N	10.91 ± 2.313	14.42 ± 2.033	0.0012*
Post Yield Displacement	mm	0.7524 ± 0.3934	0.5755 ± 0.1317	0.4385+
Work to Fracture	kJ/mm^3	10.04 ± 3.783	10.91 ± 2.320	0.5191*

Supplemental Table 9: Trabecular architecture and mechanical properties of the sixth lumbar vertebral body from placebo- and PLX5622-treated, ang II-infused mice. P-values marked with * were calculated using unpaired t test and + were calculated using Mann-Whitney test. Standard deviation is provided.

Trabecular Architecture Properties				
Property	Unit	Placebo (n=11)	PLX5622 (n=12)	P-value
Separation	μm	184.9 ± 15.74	169.8 ± 9.272	0.0091*
Connectivity Density	mm^{-3}	212.0 ± 28.97	204.0 ± 27.78	0.4966*
Structure Index	0:plate; 3:rod; 4:sphere	0.4774 ± 0.3287	-0.1220 ± 0.3532	0.0003*
Bone Area	mm^2	0.7422 ± 0.06516	0.8569 ± 0.8598	0.0013*
Compression Testing				
Stiffness	N/mm	119.4 ± 44.84	162.4 ± 80.90	0.1214*
Yield Force	N	24.48 ± 7.358	33.40 ± 9.798	0.0195*
Post Yield Displacement	mm	0.2717 ± 0.09733	0.1835 ± 0.1095	0.0535*
Axial Stress	MPa	35.25 ± 8.498	43.62 ± 6.871	0.0174*

Supplemental Table 10: Differentially expressed genes in the bone marrow of hypertensive mice treated with placebo or PLX5622. Sample size of n=5.

**Differentially Expressed Genes: PLX5622 vs. Placebo (Baseline)**		
**Gene**	**Log2(Fold Change)**	**P-value**
Ppargc1b	2.5	0.00099
Csf1	1.04	0.00191
Pknox1	1.11	0.00623
Pdgfrb	1.86	0.00778
Car2	-1.57	0.00966
Dbp	1.5	0.00974
Klf4	0.757	0.0128
Bmp6	1.21	0.0147
Hmgb1	-0.749	0.0163
Postn	1.07	0.0196

Supplemental Table 11: Antibodies used for flow cytometry.

Target Antigen	Color	Vendor or Source	Catalog #	Working Concentration
**Panel 1**				
Live/Dead	Aqua	ThermoFisher	L34957	1:100 (per 10^6)
CD45	Pacific Orange	Invitrogen	MCD4530	1:100 (per 10^6)
CD3ε	FITC	Biolegend	100306	1:100 (per 10^6)
CD4	BV 785	Biolegend	100453	1:100 (per 10^6)
CD8	PE-Cy7	Biolegend	100722	1:100 (per 10^6)
CD64	PE-Dazzle 594	Biolegend	139319	1:100 (per 10^6)
γδ TCR	APC-Cy7	Biolegend	118144	1:100 (per 10^6)
MerTK	BV 711	Biolegend	151515	1:100 (per 10^6)
I_Ab	Pacific Blue	Biolegend	116422	1:100 (per 10^6)
CD11c	PE-Cy 5	Biolegend	117316	1:100 (per 10^6)
CD115	Alexa Fluor 647	Biolegend	135530	1:100 (per 10^6)
IL17A	APC	Biolegend	506916	1:100 (per 10^6)
**Panel 2**				
Live/Dead	NIR	ThermoFisher	L34976	1:100 (per 10^6)
CD45	Alexa Fluor 700	ThermoFisher	56-0451-82	1:100 (per 10^6)
CD3	Pacific Blue	Biolegend	100214	1:100 (per 10^6)
CD19	Pacific Blue	Biolegend	152416	1:100 (per 10^6)
CD11b	BV570	Biolegend	101233	1:100 (per 10^6)
Gr-1	BV480	BD Biosciences	RB6-8C5	1:100 (per 10^6)
Sca-1	Alexa Fluor 488	Biolegend	108116	1:100 (per 10^6)
c-KIT	PE	Biolegend	105807	1:100 (per 10^6)
CD16/32	BV510	Biolegend	156625	1:100 (per 10^6)
CD34	PE-Cy5	Biolegend	119311	1:100 (per 10^6)
CD11c	BV785	Biolegend	117335	1:100 (per 10^6)
I_Ab	PE-Cy7	Biolegend	116419	1:100 (per 10^6)
CD64	PE-Dazzle 594	Biolegend	139319	1:100 (per 10^6)
MerTK	BV 711	Biolegend	151515	1:100 (per 10^6)
Ccr2	BV650	Biolegend	150613	1:100 (per 10^6)
CD115	BV605	Biolegend	135517	1:100 (per 10^6)
PU.1	Alexa Fluor 647	Biolegend	681303	1:100 (per 10^6)

Supplemental Table 12: Taqman probes for qPCR.

Name	Gene	Chromosome Location	Assay ID
Beta Actin	*Actb*	Chromosome 5: 142903116 - 14290674	Mm02619580_g1
Colony Stimulated Factor 1	*Csf1*	Chromosome 3: 107741048 - 107760469	Mm00432686_m1
Receptor Activator of Nuclear factor κβ Ligand	*Tnfsf11*	Chromosome 14: 78277446 - 78308042	Mm00441906_m1
Tartrate Resistant Acid Phosphatase	*Acp5*	Chromosome 9: 22126727 - 22135746	Mm00475698_m1
Cathepsin K	*Ctsk*	Chromosome 3: 95499210 - 95509387	Mm00484039_m1
Sclerostin	*Sost*	Chromosome 11: 101962458 - 101967015	Mm00470479_m1
Osteoprotegerin	*Tnfrsf11b*	Chromosome 15: 54250619 - 54278484	Mm00435454_m1
Runt-Related Transcription Factor 2	*Runx2*	Chromosome 17: 44495986 - 44814831	Mm00501584_m1
Alkaline Phosphatase	*Alpl*	Chromosome 4: 137741731 - 137796384	Mm00475834_m1
Dentin Matrix Protein 1	*Dmp1*	Chromosome 5: 104197558 - 104214109	Mm01208363_m1
Collagen, Type 1, Alpha 1	*Col1a1*	Chromosome 11: 94936270 – 94951867	Mm00801666_g1
Colony Stimulating Factor 1 Receptor	*Csf1r*	Chromosome 18: 61096808-61131139	Mm01266652_m1
Nuclear Factor of Activator T Cells 1	*Nfatc1*	Chromosome 18: 80606205 - 80713071	Mm01265944_m1

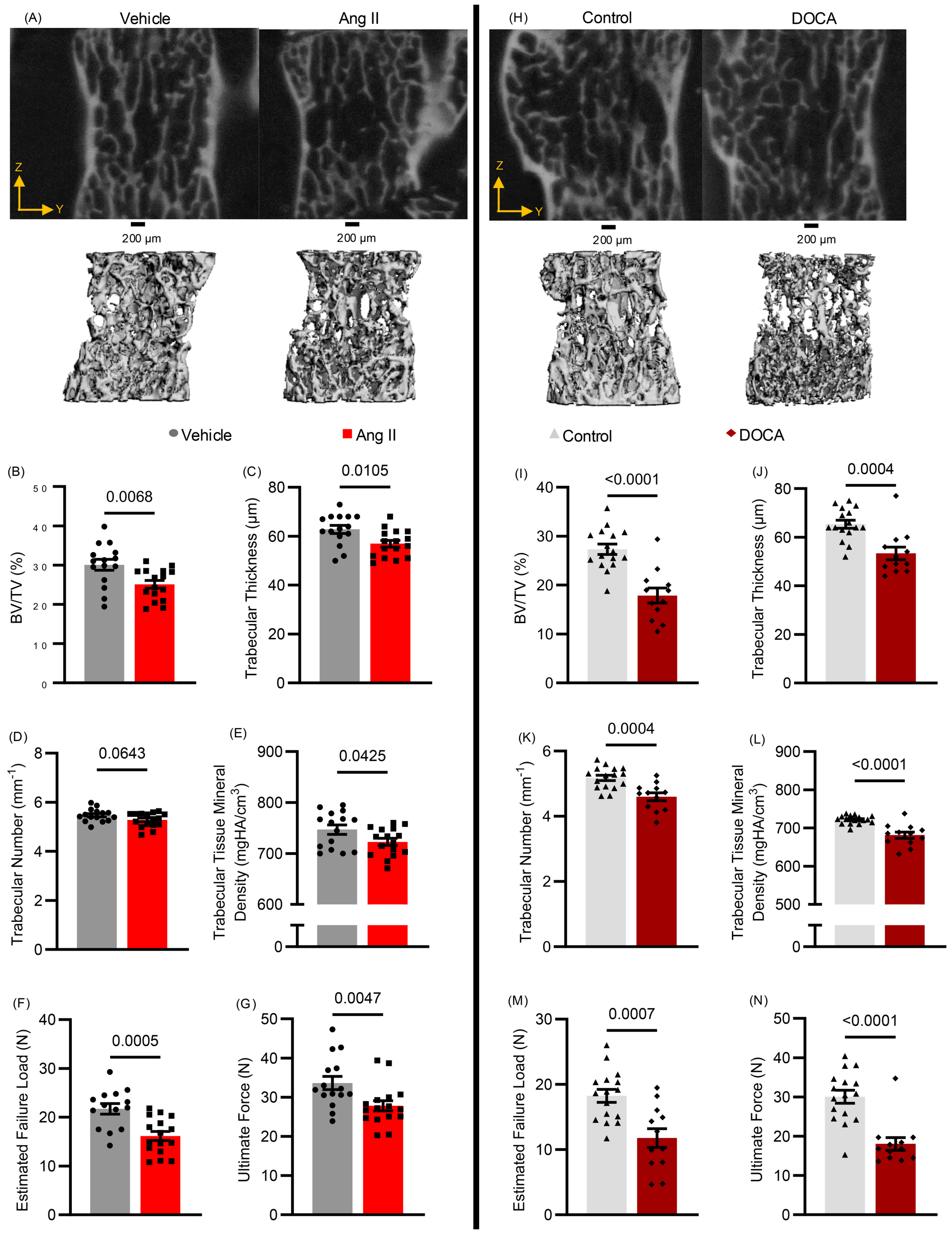

Supplemental Figure 1: Trabecular architecture of the L6 vertebral body from vehicle- and ang II-infused or control and DOCA-salt mice. Panels A and H are representative µCT 2D (top) and 3D (bottom) images of the L6 vertebrae. Panels B-G and I-N are µCT-derived parameters for BV/TV (Panels A and I), trabecular thickness (Panels C or J), trabecular number (Panels D and K), trabecular tissue mineral density (Panels E and L), estimated failure load (Panels D and I), and ultimate force (Panels E and J). Panels B-F, I-K, M and N were analyzed by unpaired T test. Panels G and L were analyzed by Mann-Whitney test. SEM is shown. Sample size: Vehicle n=15, Ang II n=16, Control n=16, and DOCA n=12.

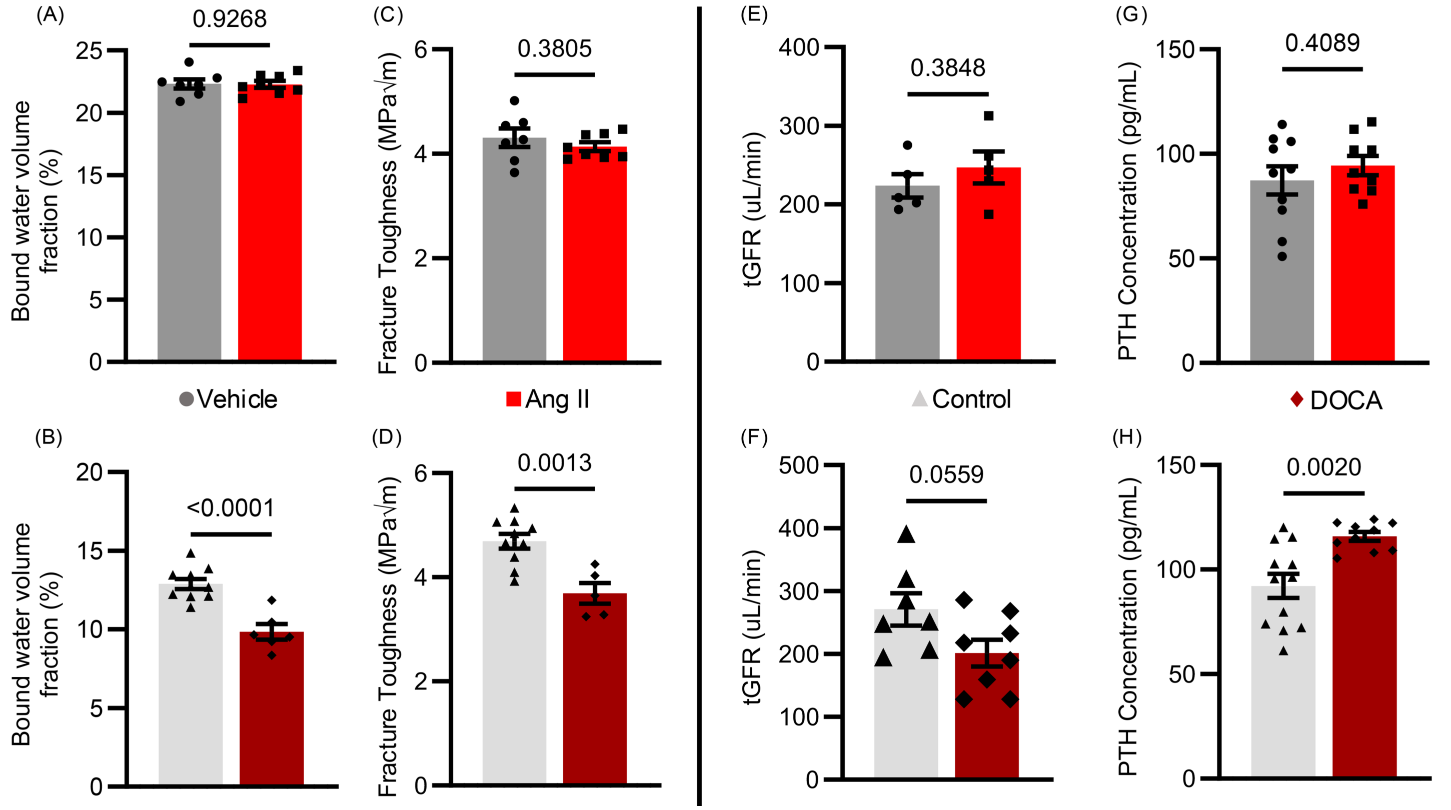

Supplemental Figure *2:* Bound water and fracture toughness of femurs from vehicle- and ang II-infused or control and DOCA-salt mice. Panels A, C, E, and G compare vehicle- and ang II-infused mice. Panels B, D, F, and H compare control and DOCA-salt mice. Panels A and B quantify bound water measurements. Panels C and D quantify fracture toughness. Panels E and F quantify tGFR. Panels G and H quantify PTH concentration in the serum. Unpaired T-test was for Panels A-G. Mann-Whitney was used for Panel H SEM is shown. Sample size: Vehicle n=5-7, Ang II n=5-8, Control n=7-12, DOCA n=5-12.

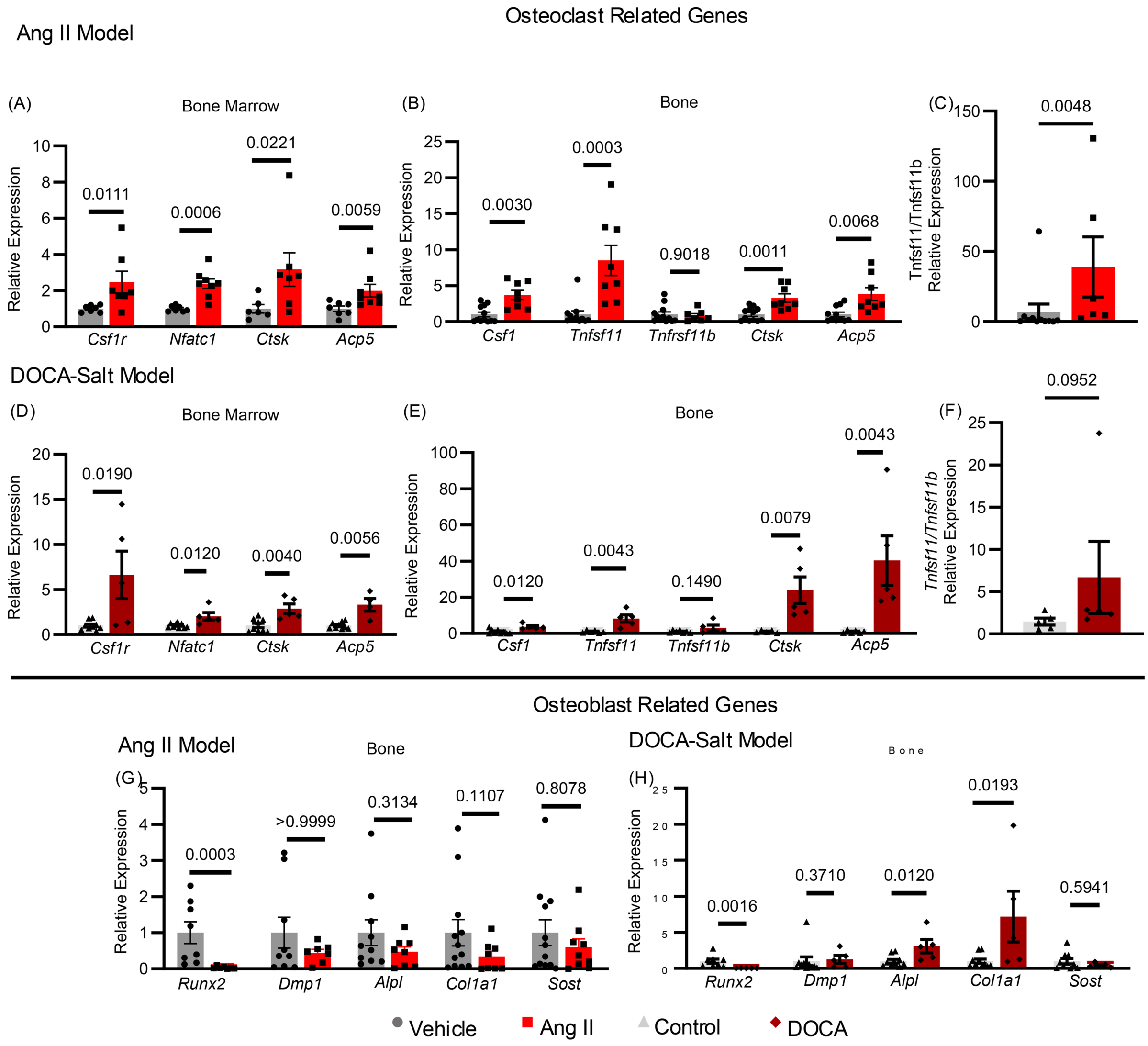

Supplemental Figure 3: mRNA expression from the bone marrow and bone of vehicle- and ang II-infused mice or control and DOCA-salt mice. Panels A-C and G are mRNA expression from vehicle- and ang II-infused mice in which Panel A is from the bone marrow, and Panels B-C and G are from the bone. Panels D-F and H are mRNA expression from control or DOCA-salt mice in which Panel D is from the bone marrow, and Panels E-F and H are from the bone. Panel A-F were analyzed by Mann-Whitney Test. SEM is shown. Sample size: Vehicle n=7-10, Ang II n=7-8, Control n=5-9, DOCA n=4-6.

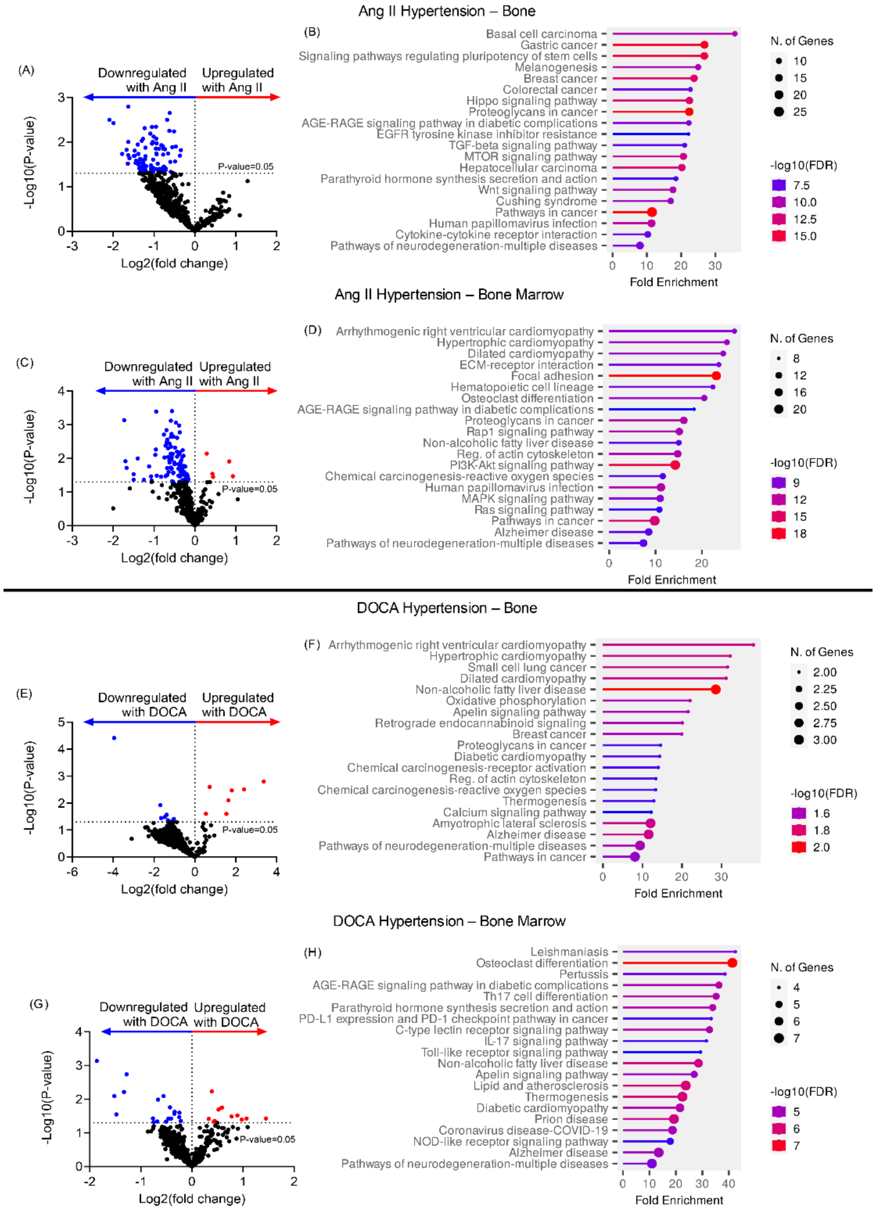

Supplemental Figure 4: Differentially expressed genes in the bone and marrow from two models. Panels A and C are volcano plots from the bone (A) and marrow (B) of Ang II infused mice compared to vehicle mice. Panels B and D are KEGG analyses of all significantly different genes in the Ang II model from the bone (B) or marrow (D). Panels E and G are volcano plots from the bone (E) and marrow (G) of DOCA mice compared to control mice. Panels F and H are KEGG analyses of all significantly different genes in the DOCA model from the bone (F) or marrow (H). Sample size: Vehicle vs. Ang II Bone n=11-12, Vehicle vs. Ang II Bone marrow n=5, Control vs. DOCA Bone n=6-9, Control vs. DOCA Bone marrow n=6-9. Abbreviations: AGE-RAGE – advanced end-products-receptor for AGE; EGFR – epidermal growth factor receptor; TGF – transforming growth factor; MTOR – mechanistic target of rapamycin; Wnt – wingless-related integration site; ECM – extracellular matrix; PI3K-Akt – Phosphatidylinositol 3-kinase-protein kinase B; MAPK – mitogen-activated protein kinases; Th17 – Helper T cell 17; PD-L1 – Programmed cell death ligand 1; PD-1 – Programmed cell death 1; NOD – nucleotide-binding oligomerization domain.

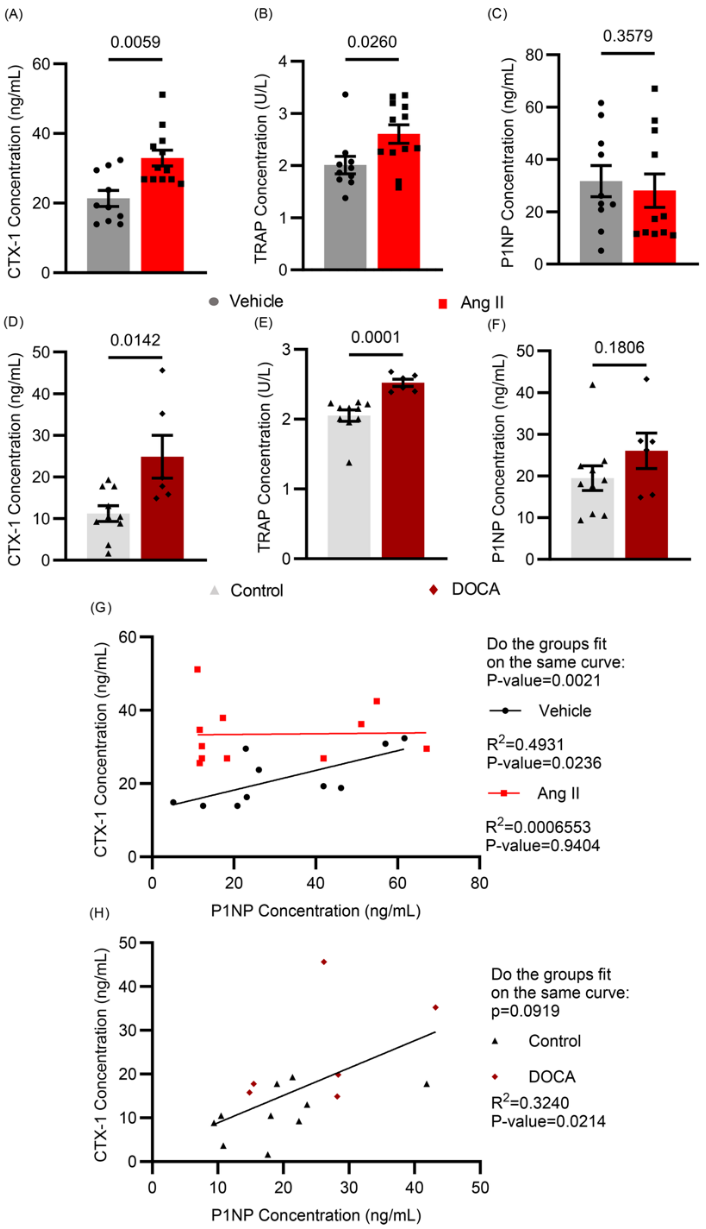

Supplemental Figure 5: Serum markers for bone remodeling in vehicle- and ang II-infused or control and DOCA-salt mice. Panels A-C and G are from vehicle- and ang II-infused mice. Panels D-F and H are from control and DOCA-salt mice. Panels A and D are for CTX-1 concentration. Panels B and E are for TRAP concentration. Panels C and F are for P1NP. Panels G and H correlate P1NP and CTX-1. For Panels A, C and E-F, Mann-Whitney was used. Panel B used unpaired T test. Panels G and H were analyzed by nonlinear regression. SEM is shown. Sample size: Vehicle n=10, Ang II n=11-12, Control n=10, DOCA n=6.

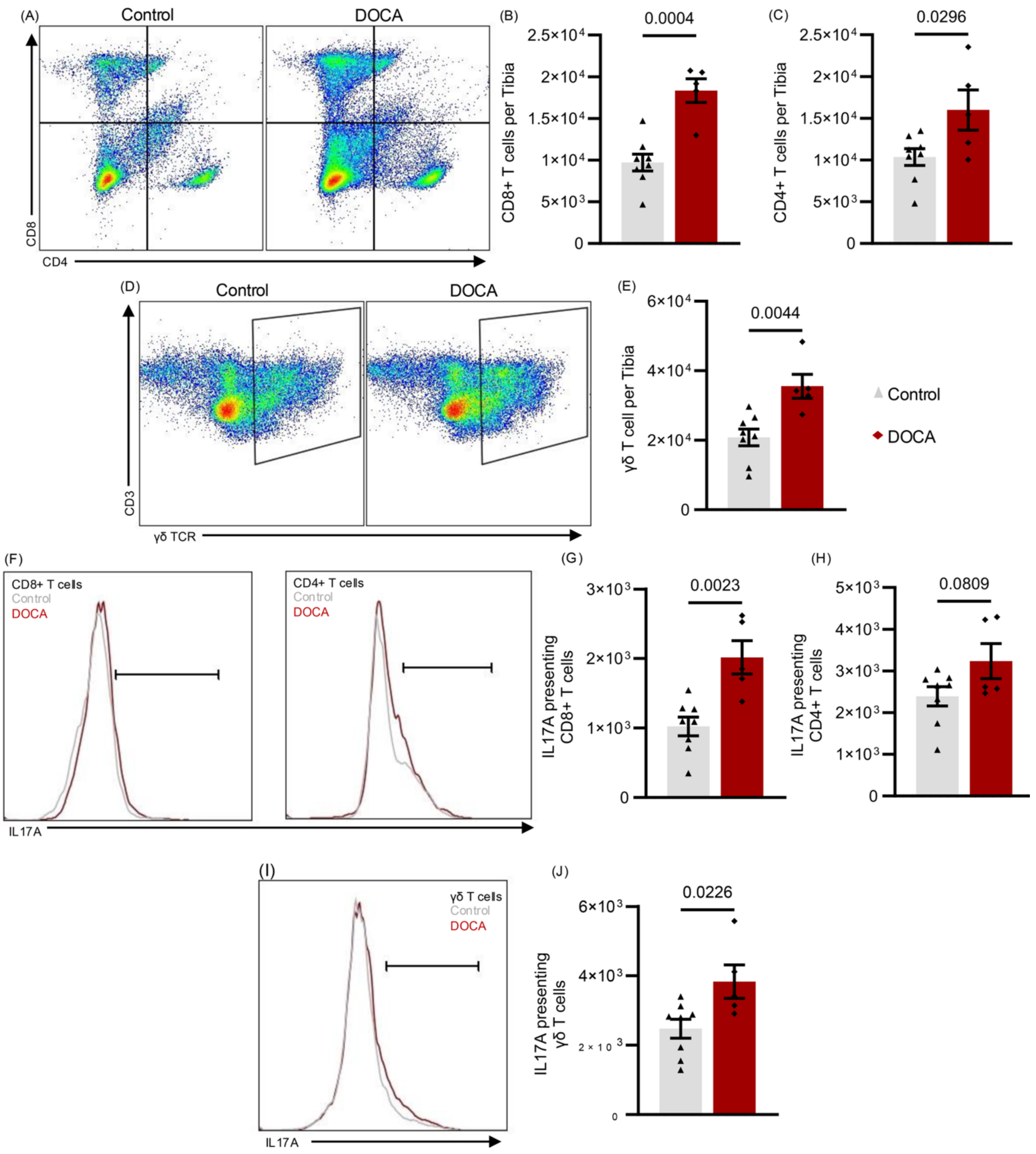

Supplemental Figure 6: Flow cytometric analysis of T cells and their activation in the bone marrow. Panels A, D, F, and I are representative images of gating for T cell subtypes (Panels A and D) or IL17A by T cell subtypes (Panels F and I). Panels B, C, and E are the quantification of T cell subtypes in the bone marrow. Panels G, H, and J are the quantification of IL17A presentation by T cell subtypes in the bone marrow. Panels B-C, E, G-H, and J were analyzed by unpaired T test. SEM is shown. Sample size: Control n=8 and DOCA n=5.

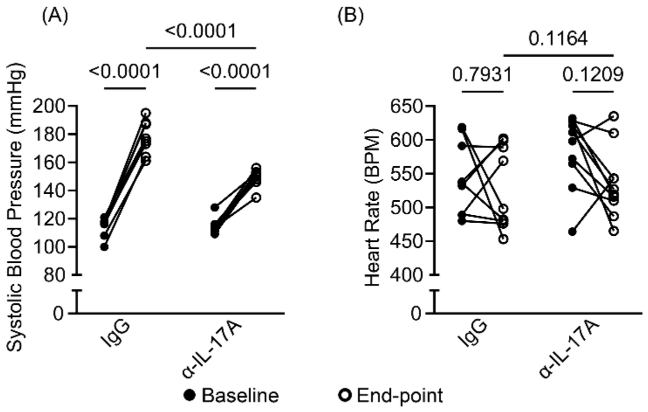

**Two-way-ANOVA Table**	P-value	F-value
Systolic Blood Pressure		
Time	<0.0001	749.0
Treatment	0.0001	24.26
Interaction	<0.0001	62.59
Heart Rate		
Time	0.0863	3.315
Treatment	0.1946	1.824
Interaction	0.3680	0.8554

Supplemental Figure 7: Blood pressure and heart rate measurements from IgG- and α-IL-17A-treated hypertensive mice. Panel A is the systolic blood pressure measurements from mice at baseline and end-point. Panel B is the heart rate measurements from mice at baseline and end-point. Two-way ANOVA table reports statistics for Panel A and B. Samples size: IgG n=9 and α-IL-17A n=10.

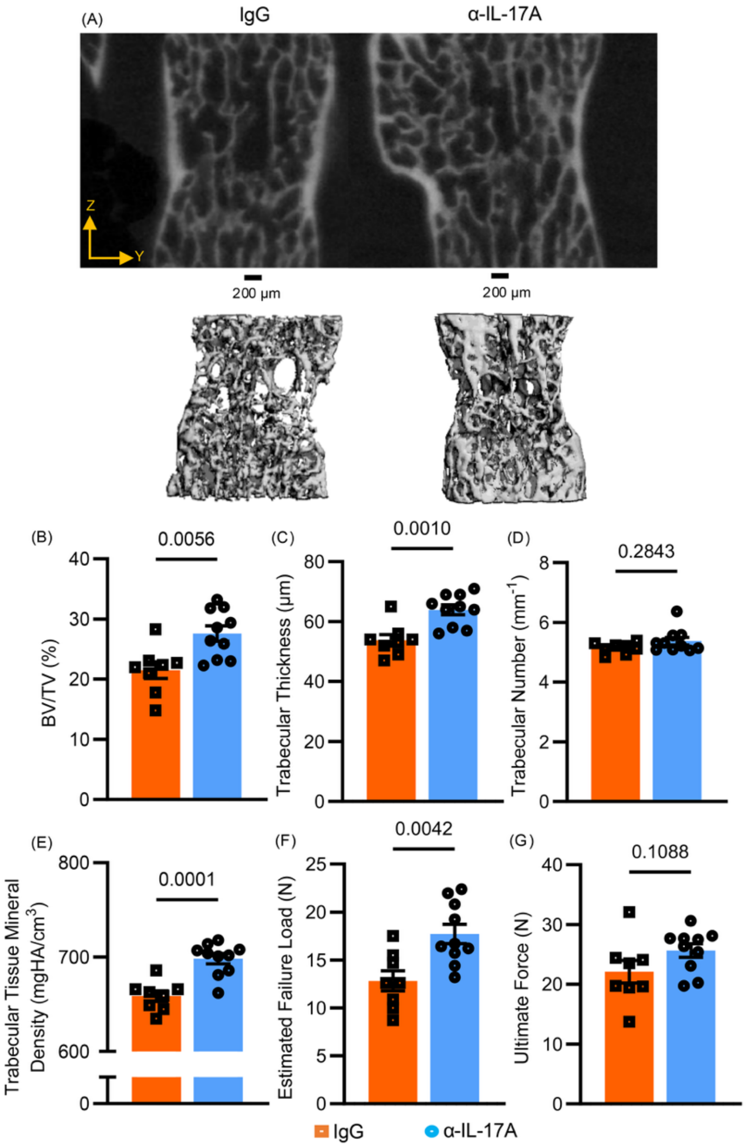

Supplemental Figure 8: Trabecular architecture of the L6 vertebral body from ang II-infused mice treated with IgG or α-IL-17A. Panels A and F are representative μCT 2D (top) and 3D (bottom) images of the L6 vertebrae. Panels B-G are μCT-derived parameters for BV/TV (Panel B), trabecular thickness (Panel C), trabecular number (Panel D), trabecular tissue mineral density (Panel E), estimated failure load (Panel F), and ultimate force (Panel G). Panels B, C and E-G were analyzed by unpaired T test. Panel D was analyzed by Mann Whitney test. SEM is shown. Sample size: IgG n=8 and α-IL-17A n=10.

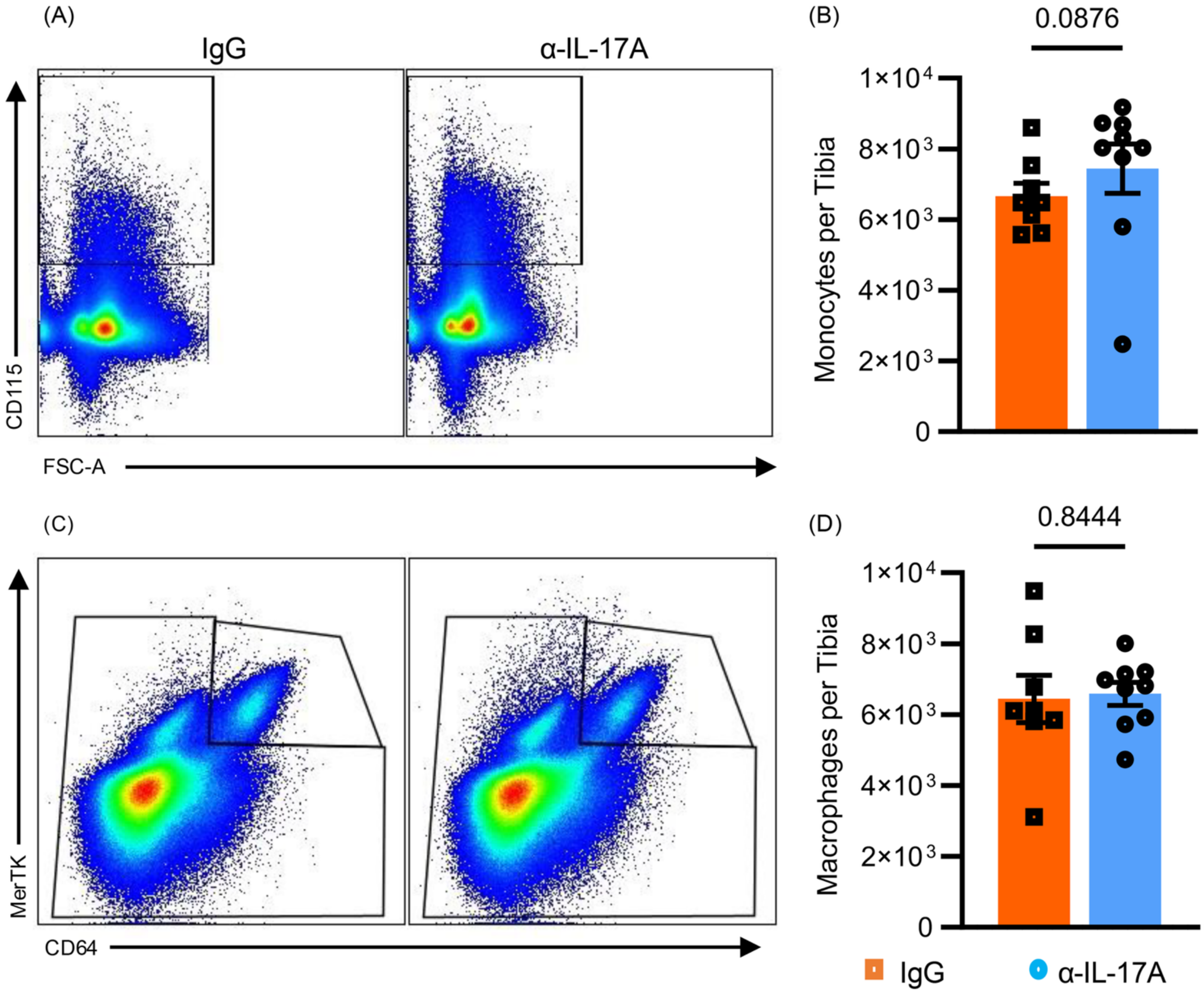

Supplemental Figure 9: Flow cytometric analysis of the bone marrow from ang II-infused mice treated with IgG or α-IL-17A for monocytes and macrophages. Panel A is the representative gating for monocytes. Panel C is the representative gating for macrophages. Panels B and D quantify the number of monocytes or macrophages, respectively. Mann-Whitney test was used. SEM is shown. Sample size: IgG n=7 and α-IL-17A n=9.

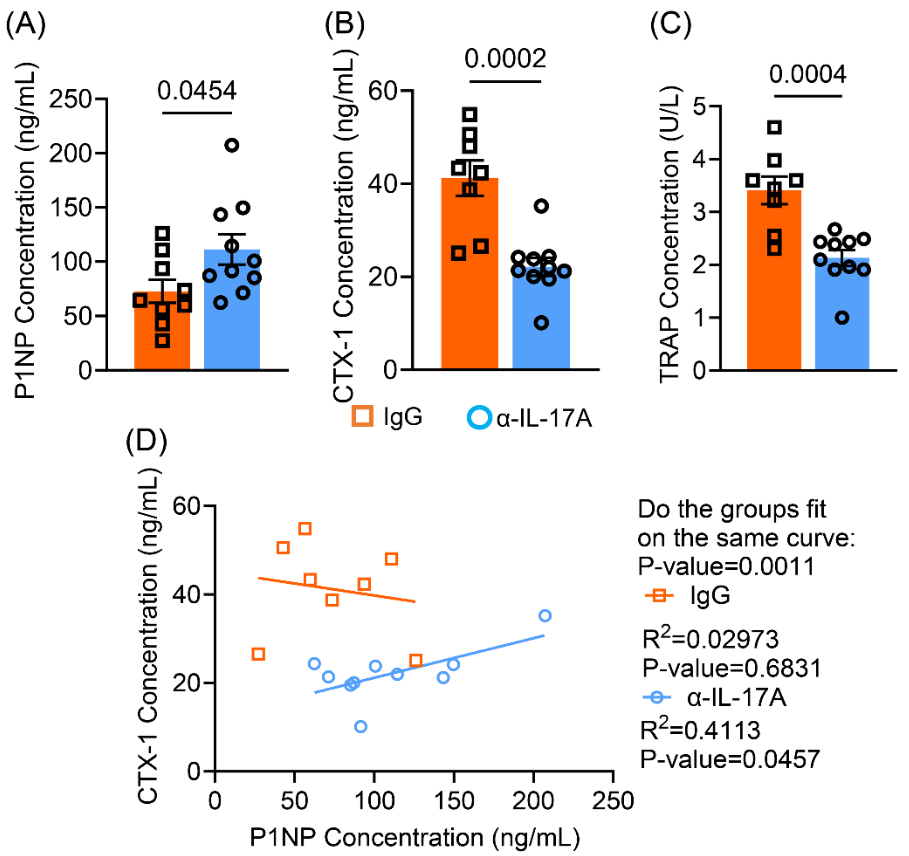

Supplemental Figure 10: Serum markers for bone remodeling in ang II infused mice treated with IgG isotype or α-IL-17A. Panel A is for P1NP concentration. Panel B is for CTX-1 concentration. Panel C is for TRAP concentration. Panel D correlates P1NP and CTX-1 using nonlinear regression. For Panels A-C, unpaired T test was used. SEM is shown. Sample size: IgG n=8-9, α-IL-17A n=10.

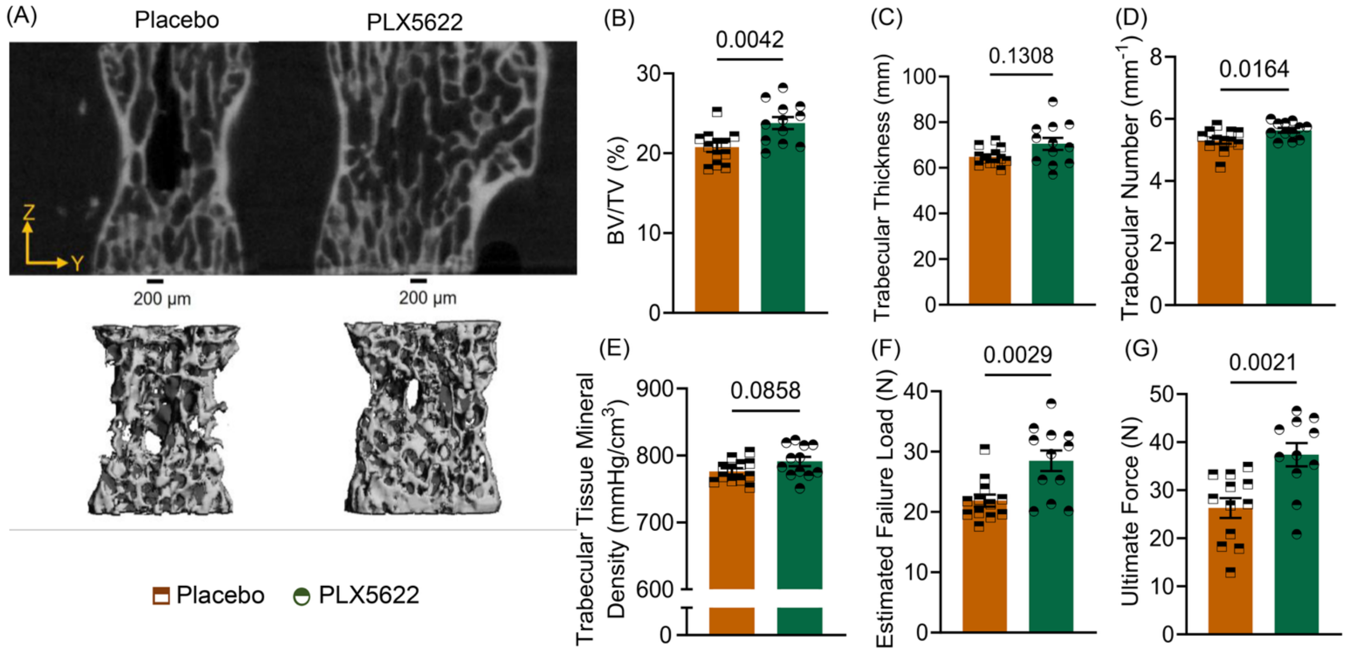

Supplemental Figure 11: Trabecular architecture of the L6 vertebral body from ang II-infused mice treated with placebo or PLX5622. Panels A are representative µCT 2D (top) and 3D (bottom) images of the L6 vertebrae. Panels B-F are µCT-derived parameters for BV/TV (Panels B and I), trabecular thickness (Panels C), trabecular number (Panels D), trabecular tissue mineral density (Panels E), and estimated failure load (Panels F). Compression tested yielded the ultimate force of the L6 vertebrae (Panels G). Panels B and D-G were analyzed by unpaired t-test. Panel C was analyzed by Mann Whitney test. SEM is shown. Sample size: placebo n=12, and PLX5622 n=12.

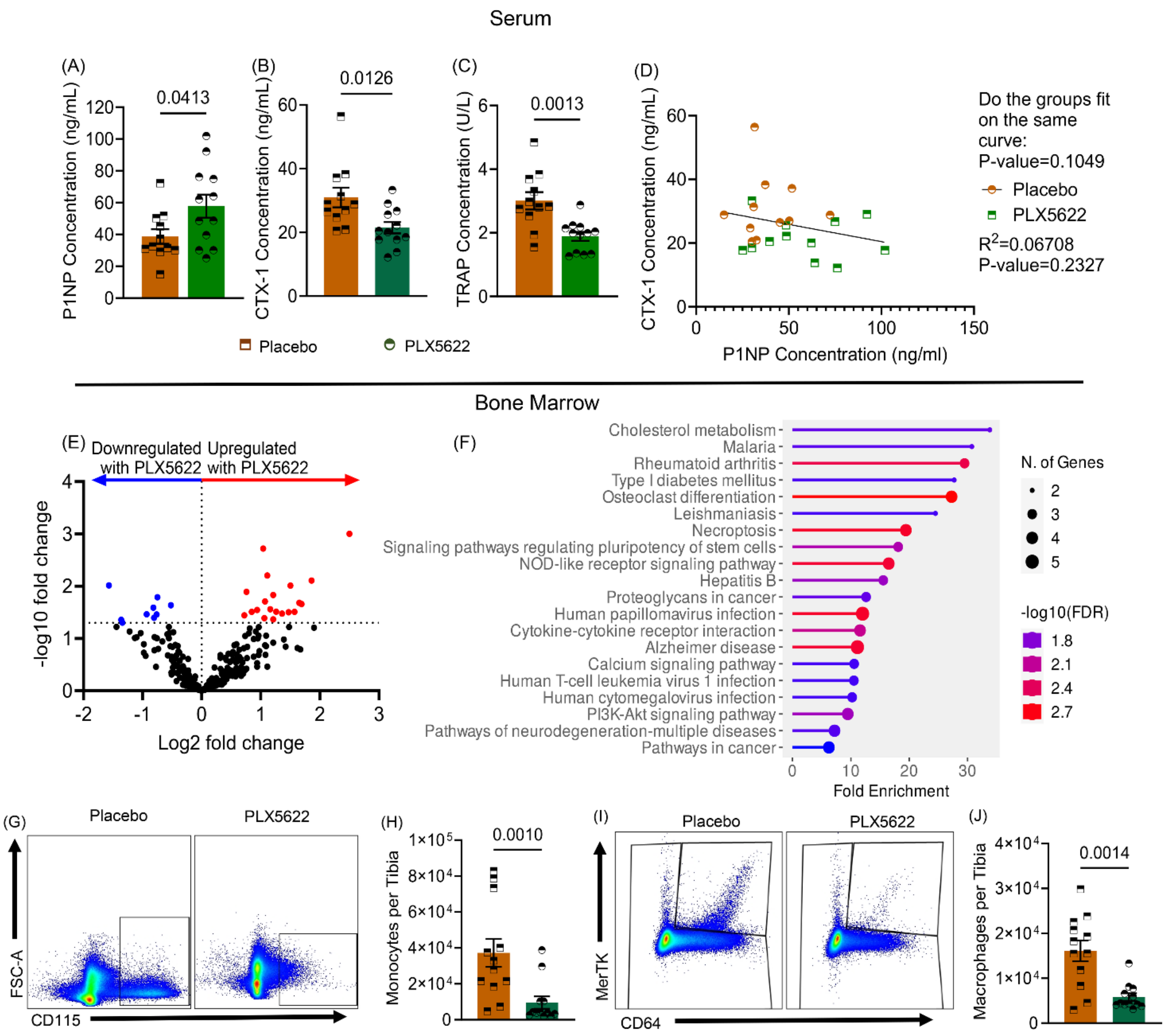

Supplemental Figure 12: Analysis of bone remodeling in ang II infused mice treated with placebo or PLX5622. Serum analysis for circulating bone remodeling markers, P1NP, CTX-1, TRAP, are in Panels A – C. Panel D correlates P1NP and CTX-1 using nonlinear regression. Panel E is the volcano plot of the differentially expressed genes in the bone marrow of PLX5622 compared to placebo treated mice. Panel F is the KEGG analysis of all significantly differentially expressed genes between placebo- and PLX5622-treated, ang II-infused mice. Panel G is the representative gating for monocytes which was quantified in Panel H. Panel I is the representative gating for macrophages which was quantified in Panel J. Panels A-C were analyzed by unpaired t-tests. Panels H and J were analyzed by Mann-Whitney tests. Panel E is the differentially expressed genes determined using Advanced Analysis program in nSolver. Panel F is the KEGG analysis done with ShinyGO 0.82 software. Sample size: placebo n=5-12, PLX5622 n=5-12.

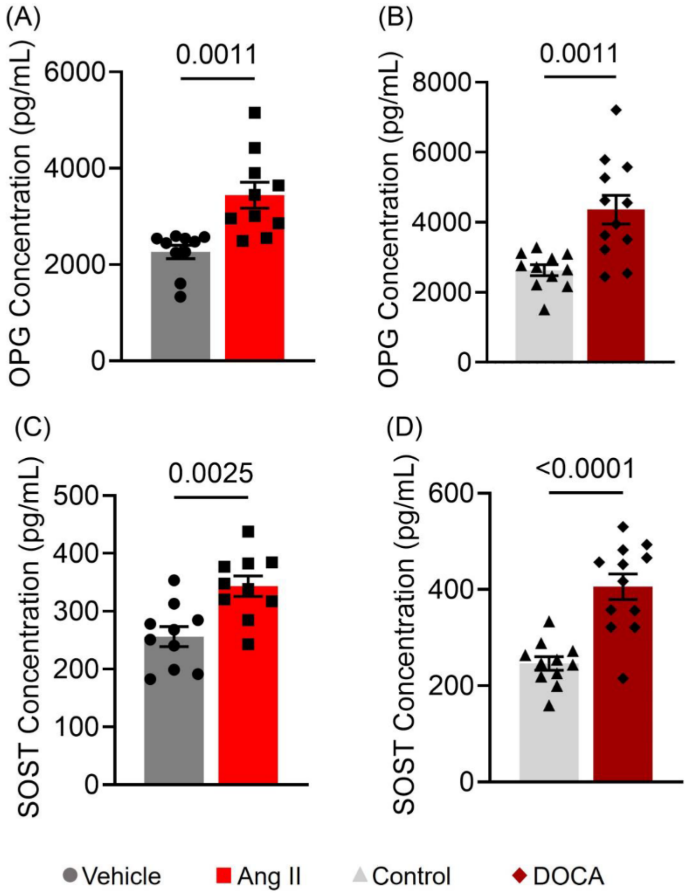

Supplemental Figure 13: Serum analysis in two models of hypertension. Panels A and C are for the ang II model of hypertension and Panels B and D are for the DOCA-salt model of hypertension. Panels A and C were analyzed by unpaired t-test. Panels B and D were analyzed by Mann-Whitney. SEM is shown. Sample size: Vehicle: n=10, Ang II: n=10, Control: n=11, DOCA: n=12.

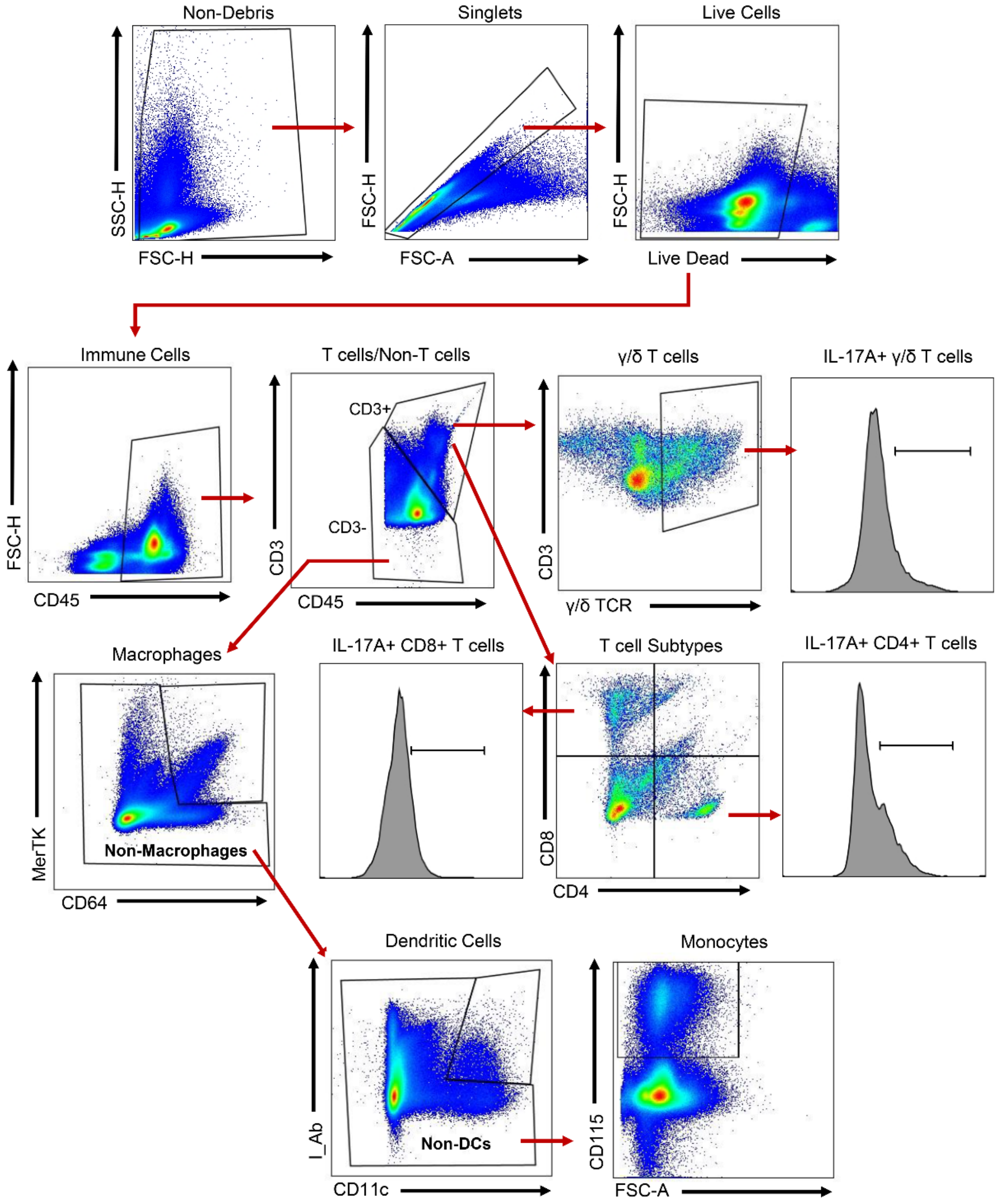

Supplemental Figure 14: Representative gating strategy for flow cytometric analysis on the bone marrow using Panel 1 as described in Supplemental Table 11.

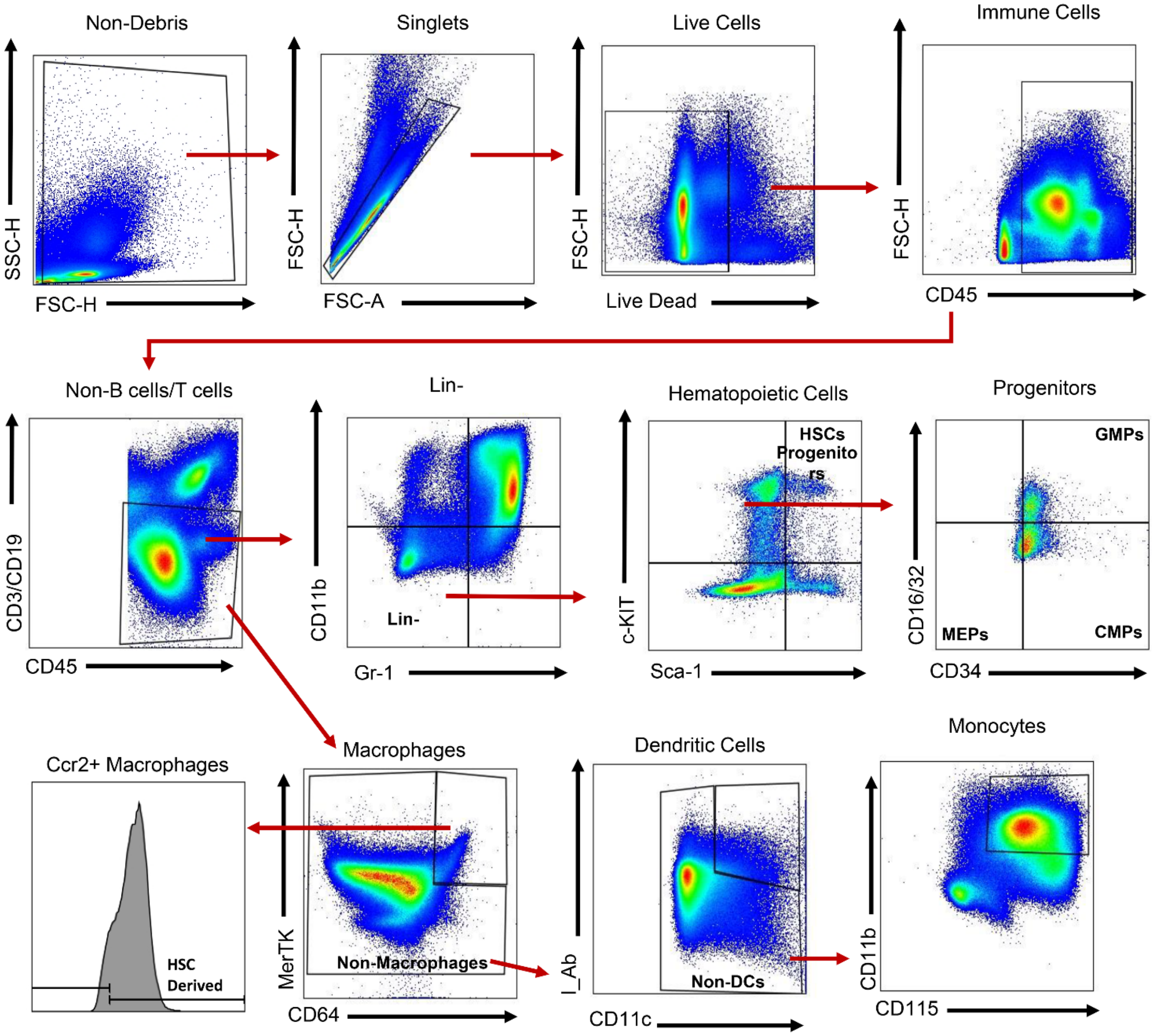

Supplemental Figure 15: Representative gating strategy for flow cytometric analysis on the bone marrow using Panel 2 as described in Supplemental Table 11.
